# Multiplex Brain Proteomic Analysis Revealed the Molecular Therapeutic Effects of Buyang Huanwu Decoction on Cerebral Ischemic Stroke Mice

**DOI:** 10.1371/journal.pone.0140823

**Published:** 2015-10-22

**Authors:** Hong-Jhang Chen, Yuh-Chiang Shen, Young-Ji Shiao, Kuo-Tong Liou, Wei-Hsiang Hsu, Pei-Hsuan Hsieh, Chi-Ying Lee, Yet-Ran Chen, Yun-Lian Lin

**Affiliations:** 1 National Research Institute of Chinese Medicine, Taipei, Taiwan; 2 Institute of Food Science and Technology, National Taiwan University, Taipei, Taiwan; 3 Department of Chinese Martial Arts and Graduate Institute of Sport Coaching Science, Chinese Culture University, Taipei, Taiwan; 4 Agricultural Biotechnology Research Center, Academia Sinica, Taipei, Taiwan; 5 Department of Chinese Pharmaceutical Sciences and Chinese Medicine Resources, China Medical University, Taichung, Taiwan; 6 School of Pharmacy, National Taiwan University, Taipei, Taiwan; North Carolina A&T State University, UNITED STATES

## Abstract

Stroke is the second-leading cause of death worldwide, and tissue plasminogen activator (TPA) is the only drug used for a limited group of stroke patients in the acute phase. Buyang Huanwu Decoction (BHD), a traditional Chinese medicine prescription, has long been used for improving neurological functional recovery in stroke. In this study, we characterized the therapeutic effect of TPA and BHD in a cerebral ischemia/reperfusion (CIR) injury mouse model using multiplex proteomics approach. After the iTRAQ-based proteomics analysis, 1310 proteins were identified from the mouse brain with <1% false discovery rate. Among them, 877 quantitative proteins, 10.26% (90/877), 1.71% (15/877), and 2.62% (23/877) of the proteins was significantly changed in the CIR, BHD treatment, and TPA treatment, respectively. Functional categorization analysis showed that BHD treatment preserved the integrity of the blood–brain barrier (BBB) (Alb, Fga, and Trf), suppressed excitotoxicity (Grm5, Gnai, and Gdi), and enhanced energy metabolism (Bdh), thereby revealing its multiple effects on ischemic stroke mice. Moreover, the neurogenesis marker doublecortin was upregulated, and the activity of glycogen synthase kinase 3 (GSK-3) and Tau was inhibited, which represented the neuroprotective effects. However, TPA treatment deteriorated BBB breakdown. This study highlights the potential of BHD in clinical applications for ischemic stroke.

## Introduction

Stroke is a devastating neurological disease and the second-leading cause of death worldwide, with stroke cases being ischemic. The World Health Organization (WHO) estimated that stroke accounted for 6.7 million deaths worldwide in 2012, which is equivalent to 11.9% of all deaths [1]. The major pathophysiological mechanism of stroke is excitotoxicity. Excessive glutamate release stimulates neurons and induces neuronal death *via* oxidative stress, the overproduction of reactive oxygen species (ROS), and the massive inflammation generated by recruited leukocytes and activated microglial cells [2]. Moreover, this acute inflammation can result in the activation of an inflammatory transcription factor (i.e., nuclear factor κB), which disrupts the blood–brain barrier (BBB) [3]. Mechanistically, phosphoinositide 3-kinase (PI3K)/Akt, and glycogen synthase kinase-3 (GSK-3) are involved in the pathology that occurs after brain ischemia. Akt plays an important role in the cell death/survival pathway. Akt phosphorylation has a neuroprotective function against ischemic injury [4]. Activated Akt can phosphorylate several downstream targets, including the constitutively active serine-threonine kinase, GSK-3 [5], which plays a key role in many fundamental processes during neurodevelopment. There are two isoforms of GSK-3, GSK-3α and GSK-3β, which have serine residues in their amino-terminal domains (Ser21 for GSK-3α and Ser9 for GSK-3β). GSK-3β is particularly abundant in the central nervous system (CNS) and is neuron specific [6]. Akt phosphorylates GSK-3 to render it inactive, which is a major mechanism *via* neurons resistant to apoptosis. GSK-3 has also been reported to be involved in ischemic brain injury [7]. The inhibition of GSK-3 rescues not only the neurogenesis, but also the hippocampal-dependent learning [8]. Conversely, increased GSK-3 activity contributes to the generation of a neuroinflammatory environment and impairs adult neurogenesis [9, 10]. These results support the idea that the inhibition of GSK-3 is a potential target in the treatment of ischemic stroke. Although the tissue plasminogen activator (TPA) was approved by the FDA as the only effective drug for a limited group of patients with ischemic stroke in the acute phase because of its narrow therapeutic timeline [11] and well-documented adverse effects, such as increased symptomatic intracerebral hemorrhage [12]. Moreover, the timing of an accurate diagnosis is essential for stroke patients, which limits the utility of the TPA treatment. Up to now, any effective treatments for stroke are yet to be developed.

Buyang Huanwu Decoction (BHD), which is a traditional Chinese medicine (TCM) prescription, has long been used to improve the recovery of the neurological function in patients with stroke by inducing neuroprotective effects against cerebral ischemia/reperfusion (CIR) injury [13–15], and to promote growth potential during peripheral neural regeneration [16, 17]. Our previous genomics study reported that BHD protects mice against ischemic stroke by downregulating inflammation-, apoptosis-, angiogenesis-, and blood coagulation-related genes, as well as upregulating neurogenesis- and the nervous system’s development-related genes [18]. Furthermore, an NMR-based metabolomics study demonstrated that BHD ameliorates the aberrant metabolism of the brain in CIR mice [19]. Hao et al., reported that BHD improves the neurological deficit and seems generally safe in patients with acute ischemic stroke, based on a clinical trial performed in China [20]. Taken together, these results suggest that BHD is efficient; it is also widely used in Chinese medicine for treating stroke. Nevertheless, the manner in which BHD protects and ameliorates the neurological function, as well as the underlying molecular mechanism from the protein perspective, remain unclear.

Proteomics is one of the fastest-growing technologies, with the farthest-reaching consequences, among the new biotechnologies developed over the past 10 years. Unlike single-compound drugs with an obvious mechanism of action, TCM is comprised of multiple components with multiple therapeutic effects on multiple targets. Stroke involves multiple cellular and molecular events, including BBB breakdown, neuronal death, neurodegeneration, etc.; thus, a single-pathway drug may not address all of these molecular mechanisms. However, it is difficult to evaluate the holistic therapeutic effects of TCM using a general-purpose technology. Moreover, most of the studies that compared therapeutic effects on ischemic stroke were mainly based on physiological (blood pressure, blood flow, oxygen level etc.) and pathological (immunohistochemical staining) observations [13–16]. Using proteomics research to screen the target molecules, evaluate the effects, or explore the mechanisms of the actions of TCM, may make up for the shortcomings of the conventional methodologies such as studying a specific single target or pathway that are often applied in the studies of TCM [21–23]. Currently, the LC-MS/MS-based multidimensional protein identification technology combined with multiplex isobaric tags for relative and absolute quantification (iTRAQ) provides a desirable approach for proteomics studies [24]. Moreover, functional proteomics combines bioinformatics, to explore and to unravel the molecular machinery of cells, with protein–protein interaction networks, which may contribute substantially to the development of molecular evidence-based TCM research [25].

In this study, an iTRAQ-based quantitative proteomics analysis was performed to characterize comprehensively the effects of BHD on ischemic stroke. To the best of our knowledge, this is the first report comparing the TCM and a conventional therapeutic approach of ischemic stroke, in cellular regulation mechanisms at the protein level. The iTRAQ analysis led to the observation of the regulation of the levels of proteins that are associated with general ischemic stroke responses, which were restored after BHD or TPA treatment. The detailed regulation mechanisms of ischemic stroke and ischemic stroke with BHD or TPA treatment at the protein level were compared and discussed. The major related proteins were further validated by western blotting. This proteomics study highlighted the ischemic proteome of the integrity of the BBB, excitotoxicity suppression, energy metabolism and neurogenesis.

## Materials and Methods

### Chemicals

Acetonitrile, formic acid, methyl methanethiosulfonate (MMTS), potassium chloride, potassium phosphate monobasic, sodium dodecyl sulfate (SDS), triethylammonium bicarbonate (TEABC), and Tris (2-carboxyethyl) phosphine hydrochloride (TCEP) were purchased from J. T. Baker (Phillipsburg, NJ). Trypsin (modified, sequencing grade) was from Promega (Madison, WI). Deionized water (18.1 μΩ•cm resistivity) from a Milli-Q system (Millipore, Bedford, MA) was used throughout this work.

### Herbal materials

BHD, Chinese herbal prescription, was prepared according to procedures reported previously [19]. Briefly, the BHD was composed of Hongqi (Hedysari Radix), Dangguiwei (Angelicae Sinensis Radix), Chishao (Paeoniae Rubra Radix), Chuanxiong (Chuanxiong Rhizoma), Taoren (Persicae Semen), Honghua (Carthami Flos), and Dilong (Pheretima), which were mixed in order at a ratio of 120:10:10:10:10:10:4.5. All materials were purchased from a local supplier and identified by Lee, I-Jung, Ph.D., the leader of the herbarium of the National Research Institute of Chinese Medicine (NRICM). The BHD was prepared by boiling in distilled water at 100°C for 30 minutes, twice. The drug suspension was lyophilized, and performed HPLC fingerprinting (S1 Fig). The drug powder was dissolved in normal saline to a final concentration of 2.0 g/mL (equivalent to the dry weight of the raw materials) for animal administration. The voucher specimens were deposited at the herbarium of the NRICM.

### Ethics statement

All animal procedures and protocols were performed in accordance with *The Guide for the Care and Use of Laboratory Animals* (NIH publication, 85–23, revised 1996) and were reviewed and approved by the Animal Research Committee at National Research Institute of Chinese Medicine. IACUC protocol no: P-99-11; IACUC Approval No: A-99-1. All surgery was performed under anesthesia, and all efforts were made to minimize suffering. We always use humane endpoints (weight loss of greater than 20% and/or loss of ability to ambulate checked every 12h) and euthanize animals prior to the end of our experiments. In this study, all data were collected at day one after stroke. At that time, all mice were survival. However, with time goes, the stroke mice showed feeble, loss of ability to ambulate and even could not eat and drink. These mice were sacrificed under a deeper euthanize in accordance the human endpoints.

### Animals and induction of middle CIR injury

Six-week-old male ICR mice were purchased from the National Laboratory Animal Breeding and Research Center, Taipei, Taiwan. The mice were housed individually and fed a laboratory standard diet (Lab Rodent Chow Die 5001, Ralston Purina Co. St. Louis, Mo) *ad libitum*. The induction of CIR injury, similar to the induction of middle cerebral artery occlusion (MCAO), was achieved using a previously reported method [19]. They are in accordance with the Stroke Therapy Academic Industry Roundtable (STAIR) criteria [26]. Briefly, mice (28–30 g) were anesthetized with a mixture of isoflurane (1.5% to 2%), oxygen, and nitrogen, and all efforts were made to minimize suffering, and performed humane endpoints evaluation every 12h after stroke induction and euthanized animal prior to the end of our experiments. A fiber optic probe was glued to the parietal bone 2 mm posterior and 5 mm lateral to the bregma, and then connected to a laser-Doppler flowmeter (MBF3, Moor Instruments Ltd., Millwey, Axminster, UK) for continuous monitoring of the cerebral blood flow (CBF). For right middle cerebral artery (RMCA) occlusion in mice, a heat-blunted monofilament surgical suture (6–0) was inserted into the exposed external carotid artery, advanced into the internal carotid artery, and wedged into the Circle of Willis to obstruct the origin of the RMCA. The filament was left in place for 30 min and then withdrawn. Only animals that exhibited a reduction in CBF >85% during RMCA occlusion and a CBF recovery >80% after 10 min of reperfusion were included in this study. The average successful rate of the surgery for the induction of ischemic stroke is around 80%. This procedure leads to reproducible infarcts that are similar in size and distribution to those reported by other researchers using transient RMCA occlusion of comparable duration [27]. Rectal temperature was monitored and kept constant (37.0°C ± 0.5°C) during the surgical procedure and the recovery period, until the animal regained full consciousness.

### Animal grouping and drug treatment

Thirty-six mice were randomly divided into four groups: (i) sham control (n = 9); (ii) CIR (n = 9); (iii) BHD treatment (CIR+BHD; 1.0 g/kg, p.o., twice daily) (n = 9); and (iv) TPA treatment (CIR+TPA; 10 mg/kg, i.v. by tail vein at day one) (n = 9). At 2-h interval after the CIR induction, the mice were treated with the first dose of an appropriate drug (CIR+BHD), or vehicle control (CIR and sham groups) daily. The next dose was administered every 12h for BHD. The dose of TPA (10 mg/kg, i.v.) was transferred from human to mouse by FDA-approved equation based on the body surface area [28]. All animals were allowed to move, drink, and take food freely. Additional animals (n = 36), grouped as described above, were used for the analysis of brain infarction and for immunohistochemical staining. The detail of the experiment procedure is as shown in S2 Fig.

### Assessment of neurological deficit of mice

The neurological deficit of mice was evaluated on day 1 (24 h later) after ischemic stroke by analyzing their tracking distance and typical tracking pattern (circling behavior) within three min in a behavior observation box (60 × 40 × 60 cm) using video-tracking system software (SMART v2.5.21, Panlab, Spain). For the evaluation of the “typical” tracking pattern (circling clockwisely or so-called “chasing tail”) of the ischemic stroke mice, we performed this improved method (“open field test”) by analyzing their tracking distance and the typical tracking pattern (circling behavior) within 3 min in a behavior observation box (60×40×60 cm^3^) using a *v*ideo-tracking system software (SMART v2.5.21, Panlab, Spain). This improved method is relative more sensitive and is better than our previous method (to score (0~4) the neurological deficit) for ischemic stroke.

### Positron-emission tomography (PET) evaluation of brain function

Cerebral glucose metabolism was measured to evaluate the brain function on day 1 (24 h later) after an ischemic stroke. Animals were injected with 100 μCi of 2-deoxy-2-[^18^F]fluoro-D-glucose and imaged using a small-animal PET scanner (μPET; Concorde Microsystems). Images were acquired for 10 min under inhalation anesthesia (isoflurane, 2%). The level of radioactivity in brain tissues (percentage dose/g) was estimated from the images according to the method published by Chern *et al*.,[29].

### Protein extraction

Twenty-four hours after the ischemic stroke, mice were sacrificed by rapid decapitation under deep anesthesia. The ipsilateral ischemic brain hemisphere (whole right brain) were collected from mice of different treatment groups and stored at –80°C. Frozen brains (10 mg) were thawed on ice and then resuspended in 100 μL of RIPA lysis buffer (20 mM Tris-HCl, 2 mM EDTA, 500 μM sodium orthovanadate, 10 μg/mL of aprotinin, 10 mM NaF, 1% Triton X-100, and 0.1% SDS; pH 7.4), which was followed by homogenization on ice for 20 min. The lysates were clarified by centrifugation at 10,000 × *g* for 30 min at 4°C and then stored at –80°C.

### Protein reduction, alkylation and digestion

The total proteins were further diluted to 1 μg/μL with 50 mM TEABC, reduced with 5 mM TCEP for 1 h at 37°C, followed by alkylation using 2 mM MMTS for 45 min at room temperature. For proteolytic digestion, the modified tube gel digestion protocol was applied, and the detergent residue was checked using a previously described method [30]. The solutions of 17.5 μL of acrylamide (40%, 29:1), 2.45 μL of 10% ammonium persulfate, and 1.05 μL of TEMED were sequentially added to the protein solution. After 30 min incubation at room temperature, the acrylamide was polymerized and the protein was embedded in the gel. The tube-gel was cut into small pieces and washed with 25 mM ABC (pH 8.2) containing 50% ACN for 15 min four times. To test the surfactant residue in the wash solution, which was first dried in a centrifugal concentrator (miVac Duo Concentrator, Genevac, NY), and then the VISA test was conducted. After the gel wash and VISA test, the gel pieces were dehydrated with 100% acetonitrile and digested with trypsin (1:100 trypsin to protein ratio in weight) in 25 mM ABC at 37°C overnight. After digestion, the tryptic peptides were extracted from the gel using 25 mM ABC, 0.02% TFA, 0.02% TFA in 50% ACN, and 100% ACN sequentially.

### Labeling of digested peptides with iTRAQ reagent

To label tryptic peptides with 4-plex and 8-plex iTRAQ labeling reagents (Applied Biosystems, Foster City, CA), each of the samples was reconstituted with isopropanol individually and the labeling procedure was performed according to the manufacturer’s protocol. In this study, samples from the Sham, CIR, CIR+BHD, and CIR+TPA groups in the first biological replicate were respectively labeled with reagents 114, 115, 116, and 117 from the 4-plex iTRAQ kit. Samples from the Sham, CIR, CIR+BHD, and CIR+TPA groups in the second biological replicate were respectively labeled with reagents 113, 114, 115, and 116 from the 8-plex iTRAQ kit. Samples from the Sham, CIR, CIR+BHD, and CIR+TPA groups in the third biological replicate were respectively labeled with reagents 117, 118, 119, and 121 from the 8-plex iTRAQ kit. In the iTRAQ analysis, the protein ratios for each sample reporter ion channel to reference reporter ion channel (Sham) comparison were normalized by a factor which making the log 2 of the median ratios equal to zero.

### Strong cation exchange chromatography for peptides

For strong cation exchange (SCX) fractionation, the buffers SCX-A (5 mM KH_2_PO_4_ in 25% ACN at pH 3) and SCX-B (5 mM KH_2_PO_4_ and 350 mM KCl in 25% ACN at pH 3) were used as the mobile phase. The peptide mixtures were reconstituted in buffer SCX-A and then loaded onto a PolySULFOETHYL A column (200 × 2.1 mm, 5 μm, 300 Å; PolyLC, Columbia, MD) for 10 min at a flow rate of 0.2 mL/min. Peptides were fractionated using a 75 min gradient from 0% to 100% of buffer SCX-B and monitored at OD 214 nm. Fractions were collected every minute from the retention times of 8 to 58 min using a fraction collector (BioFrac Fraction Collector, BioRad Laboratories, Hercules, CA). For the SCX analysis, after mixing the iTRAQ peptides, about 400 μg of the total peptide were loaded into the SCX column. It is difficult to determine the peptide quantity in each SCX fraction. The detail SCX chromatogram is as shown in S3 Fig. After the SCX fractionation, each of the fractions was dried and subjected to the SPE purification to remove the excess salt using ZipTip (Millipore, Darmstadt, Germany).

### LC-MS/MS analysis

LC-MS/MS analysis was performed on a nanoUHPLC system (nanoACQUITY UPLC, Waters, Millford, MA) that was coupled online to the nanoelectrospray source of a hybrid QTOF mass spectrometer (SYNAPT HDMS G2, Waters, Manchester, UK). For the LC-MS/MS analysis, water with 0.1% FA and ACN with 0.1% FA were used as the mobile phase. The sample was injected into a tunnel frit packed trap column (5 μm particles and 2 cm in length; Symmetry C18, Waters, Milford, MA) [31] and separated online using a reversed-phase column (BEH C18, 1.7 μm, 75 μm × 250 mm; Waters, Milford, MA) at a flow rate of 300 nL/min using a 95 min gradient with 1%–90% ACN/water. The MS instruments were all operated in the positive ion mode, and data-dependent acquisition methods were applied. The data-dependent acquisition settings were set to one full MS scan (400–1,600 *m*/*z*) with a scan time of 0.6 s, and switched to three product ion scans (100–1,900 *m*/*z*) with a 1.2 s scan time when the precursor ion charge was 2+, 3+, or 4+, and the intensity was higher than 1,500 counts. After Zip-Tip purification, one-third were loaded into the MS, since the capacity of the Zip-Tip is about 3 μg thus it was about 1 μg of the peptides injected into the system for each of LC-MS analysis.

### Spectrum processing

The spectra generated from the LC-MS/MS were first converted into the mzXML format using massWolf (version 4.3.1). The MS/MS spectra in the mzXML format were subjected to spectral processing using the UniQua software, version 1.1 [31]. For MS/MS spectral processing, the parameters for SYNAPT HDMS G2 were: smoothing = 7, centroiding high = 80%, maximum resolution = 20,000, and baseline cutoff = 30 counts. The processed MS/MS spectra were then extracted and converted into the Mascot generic format (.mgf) using the mzXML Search in Trans Proteomics Pipeline (TPP), version 4.4 rev. 1.

### Protein qualification

The spectra processed by UniQua were searched against the IPI mouse protein database version 3.74 (total protein entries: 56,865), using the Mascot version 2.3 (Matrix Science, London, UK) search engine. In the Mascot search, the peptide and MS/MS fragment mass tolerances were ±0.1 Da, and ±0.1 Da, respectively. Only one missed cleavage was allowed for tryptic peptides. In the first biological replicate analysis, the methylthiolation of cysteine, N-terminal of the iTRAQ 4-plex, and lysine of the iTRAQ 4-plex were set as fixed modifications, and the oxidation of methionine and tyrosine of the iTRAQ 4-plex was set as a variable modification. In the second and third biological replicate analyses, the methylthiolation of cysteine, N-terminal of the iTRAQ 8-plex, and lysine of the iTRAQ 8-plex were set as fixed modifications, and the oxidation of methionine and tyrosine of the iTRAQ 8-plex was set as a variable modification. Peptides were considered as identified if their Mascot individual ion score was a Mascot score > 20. The protein was considered as identified if two unique peptides were identified. The overall false discovery rate for the above setting was determined to be lower than 1% with the use of the ProteinProphet (*p* < 0.05) function [32] in the Trans-Proteomics Pipeline (TPP) package (Version 4.3.0, Seattle Proteome Center, WA).

### Protein quantitation

For the isobaric labeling experiment, identification results were obtained from Mascot. The criteria used for protein quantitation were: unique peptide hits ≥2, quantitative peptides ≥2, and disabling of outlier removal. The reporter ion ratio for each identified peptide was determined by Mascot, with an intensity that should be ≥30. The biological repeat included three technical repeats. The expression of proteins was considered as being significantly changed if the fold change was > 1.3, or < 0.77, and if *p* < 0.05.

### Western blotting

The protein contents of the supernatants were measured using the Bio-Rad protein assay kit. Ten micrograms of the samples was fractionated on 8% and 12% SDS–PAGE and transferred onto a PVDF membrane. The membrane was blocked with 5% nonfat milk in TBS-Tween buffer (25 mM Tris, 190 mM NaCl, and 0.5% Tween-20; pH 7.5) for 1 h at room temperature, followed by incubation overnight at 4°C with appropriate primary antibodies (S1 Table). After hybridization with the primary antibodies, the membrane was incubated with an HRP-labeled secondary antibody for 1 h. After washing with TBS-Tween buffer, the membrane was developed with enhanced chemiluminescence (ECL) western blotting reagents (Amersham Pharmacia Biotech). Immunoblot images were analyzed using ImageJ (NIH, USA).

### Immunohistochemical (IHC) staining

Twenty-four hours and 3 days after CIR, the brains were prepared for confocal imaging of the BBB breakdown and neurogenesis, respectively, as described in our previous report [29]. Six consecutive brain sections (thickness, 20 μm) from each group were cut and collected at the same rostrocaudal levels (bregma –1.5 to –1.7 mm). After fixation, permeabilization, and blocking, the brain slices were randomly selected for incubation with appropriate primary antibodies against CD11b (1:100, BiolegeCaMKnd, San Diego, CA, USA), Ca^2+^/calmodulin-dependent protein kinase II (CaMKII) (1:250), occludin (1:100), doublecortin (DCX, 1:100) (all from Abcam, Cambridge, UK), and caspase3 (1:100, Merck-Millipore) in PBS containing 3% albumin at 4°C overnight. After washing, the sections were incubated with AlexaFluor®488-, AlexaFluor®555-, or AlexaFluor®647-conjugated secondary antibodies (Cell Signaling Technology Inc., MA, USA). All cover slips were mounted with mounting medium containing 4′, 6-diamidino-2-phenylindole (DAPI) to counterstain the DNA in nuclei. The sliced tissues were examined using a laser-scanning confocal microscope (Zeiss LSM780; Carl Zeiss, Jena, Germany). The distribution and number of immunopositively stained cells were determined and quantified based on the averaged fluorescence intensity (arbitrary units) using the imaging software Zen 2011 (black edition; Carl Zeiss MicroImaging GmbH, 1997–2011) in the entire field of the selected images or after sampling of specific regions, as indicated under high magnification (60–100×), over 3–5 independent experiments.

### Statistical analysis

Results are expressed as the mean ± standard deviation (SD) of the three experiments. A two-tailed Student *t*-test was employed to assess statistical significance using the SPSS system, vers. 11.0 (SPSS, Chicago, IL, USA). A probability of *p* < 0.05 was considered to be a statistically significant difference between two groups.

## Results

### BHD provided neuronal protection in ischemic stroke mice

**Fig 1:**
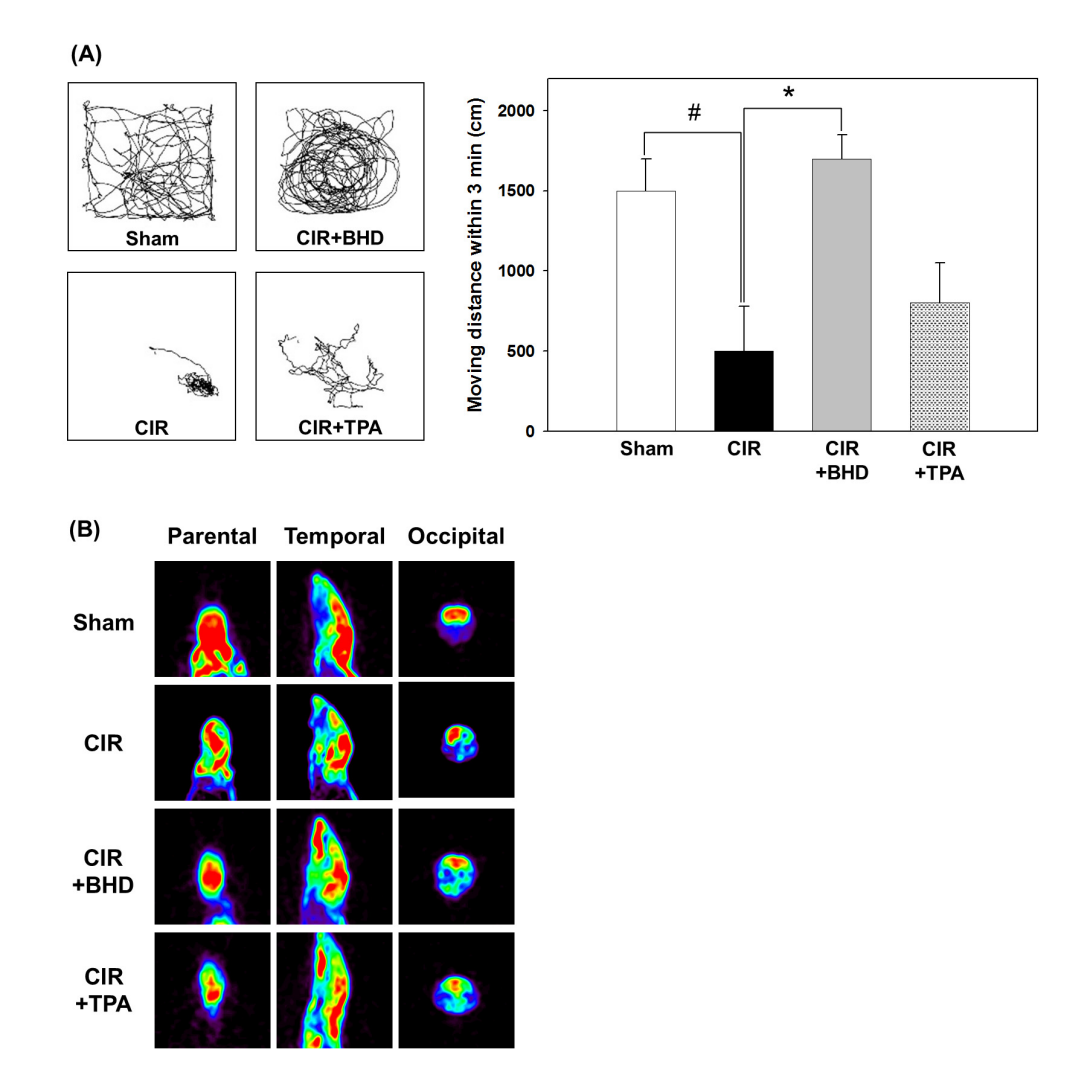
Effects of BHD on neurological deficits and brain function in mice after cerebral ischemic/reperfusion (CIR) injury. (A) Typical animal-tracking profiles within 3 min for the evaluation of neurological deficits. (B) Representative micro-PET analysis of brain function (glucose metabolism) in live mice at 24 h after stroke. Animal groups include sham, vehicle-treated animals (ischemic stroke, CIR), BHD-treated animals (CIR+BHD; 1.0 g/kg, p.o., twice daily), and TPA-treated animals (CIR+TPA; 10 mg/kg, i.v. at day one), with treatment administered 2 h after ischemic stroke. The experiment was repeated at least 3–5 times, with similar results. #*p* < 0.05 compared with the sham group; *p < 0.05 compared with the CIR group.

After BHD (1.0 g/kg, p.o., twice daily) or TPA (10 mg/kg, i.v., by tail vein at day one) treatment led to an improved the locomotor activity and behavior response in a novel open field were used to analyze neurological deficits in the CIR mice (normal saline, p.o., twice daily). As shown in Fig 1A, the CIR induction caused severe neurological deficits, which manifested as a dramatical reduction in the tracking distance and clockwise movement. Although the typical neurological deficit pattern was still observed in the TPA-treated group, it was distinctly improved in the BHD-treated group. The BHD treatment significantly increased the tracking distance (1710 ± 150 cm) to a level which was similar to that of the sham group (1520 ± 210 cm). However, the TPA treatment could not improve the locomotor activity. In addition, neurofunctional studies were carried out by determining the glucose metabolism in the brain using μPET imaging (Fig 1B). In this study, the CIR injury dramatically impaired glucose metabolism in the right brain of live mice. In contrast, treatment with BHD significantly reversed the CIR-induced damage. Taken together, our data suggests that BHD exerts its neurotherapeutic effects by improving locomotor activity, and restoring the energy metabolism in ischemic stroke mice.

### Quantitative and qualitative proteomics analysis

In this study, the brain tissue proteomes of mice that received sham, CIR, CIR with BHD treatment (CIR+BHD) and CIR with TPA treatment (CIR+TPA) were compared using an iTRAQ approach. This quantitative proteomics analysis was performed in biological triplicate. The average geometric standard deviation for bio-replicate 1, 2, and 3 were 1.209, 1.188 and 1.194, respectively. The results of the proteomics analysis are shown in S2 Table. In the first biological replicate, 92,138 MS/MS spectra were acquired and 9,863 distinct peptides were identified and translated into 1,310 proteins identified with high confidence (*p* < 0.05 and at least two unique peptides per protein). Among the 1,310 proteins identified, 1,206 were quantified because those proteins produced reporter ions with an intensity which was sufficient for reliable quantitation (average reporter ion intensity >10). In the second and third bioreplicates, using the same criteria, 88,351 spectra were acquired and 11,092 distinct peptides were identified and translated into 1,235 proteins identified with high confidence. Among the 1,235 proteins identified, 1,113 were quantified. The combination of the protein quantification results of the three biological replicates revealed the presence of 877 commonly quantified proteins in the three biological replicates. When compared with the sham group, 10.26% (90/877), 1.71% (15/877), and 2.62% (23/877), of the proteins were considered as being significantly regulated (*p* < 0.05 and fold change > 1.3) in the CIR, CIR+BHD, and CIR+TPA groups, respectively.

**Fig 2:**
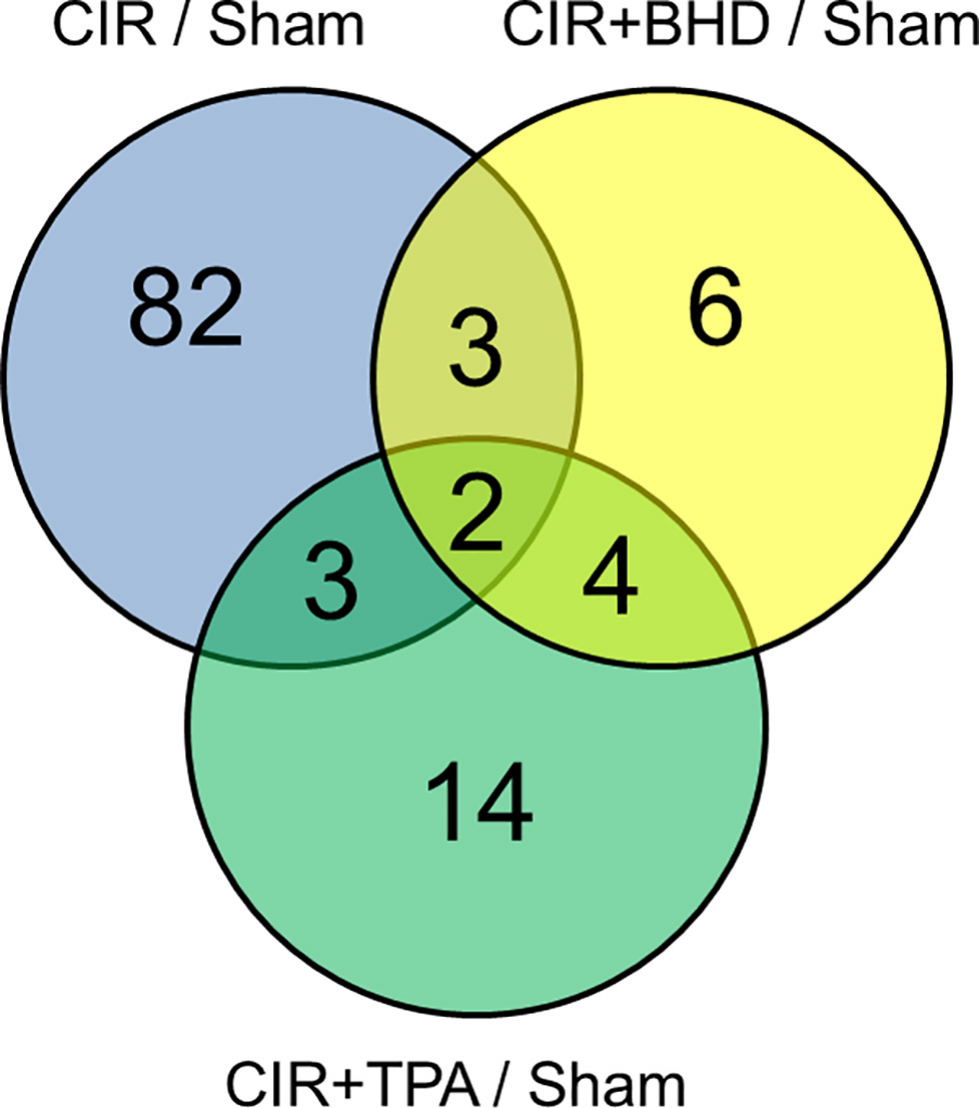
Significant changes in the protein ratios in CIR/sham, CIR+TPA/sham and CIR+BHD/sham groups according to the changes of at least 1.3-fold (*p*-value < 0.05).

Fig 2 shows a Venn diagram illustration of the number of differentially expressed proteins as a result of regulation by the three different treatments (The detail protein list was shown in S3 Table). Compared with the CIR+BHD or CIR+TPA group, the CIR group showed significant proteome changes in terms of the number of total regulated proteins. Regarding the number of proteins that were identified as being differentially regulated, there were 90 such proteins in the CIR group, but only 23 and 15 in the TPA and BHD groups, respectively. There were 82 proteins that were uniquely regulated by the CIR treatment, whereas only fourteen and six proteins were regulated by the TPA and BHD treatments, respectively. The proteins that were regulated by the TPA treatment alone included albumin (Alb), complement component 3 (C3), diazepam binding inhibitor (Dbi), dihydropyrimidinase 3 (Dpysl3), glutamyl-prolyl-tRNA synthetase (Eprs), fibrinogen (Fga), glial fibrillary acidic protein (Gfap), N-ethylmaleimide-sensitive factor attachment protein, alpha (Napa), 26S proteasome non-ATPase regulatory subunit **(**Psmd) 7, Psmd8, solute carrier family 1, member 2 (Slc1a2), solute carrier family 30 (zinc transporter), member 3 (Slc30a3), transportin 2 (Tnpo2) and transferring (Trf). The proteins that were regulated by both CIR and CIR+TPA included catalase (Cat), clusterin (Clu) and dihydropyrimidinase-like 5 (Dpysl5). The proteins that were only regulated by CIR+BHD treatment included drebrin 1 (Dbn1), endoplasmic reticulum resident protein 44 (Erp44), gamma-aminobutyric acid receptor subunit beta-3 (Gabrb3), hemoglobin alpha, adult chain 1 (Hba-a1 or Hba-a2), thymus cell antigen 1, theta (Thy1) and RIKEN cDNA 4930572J05 gene (Them6). Moreover, the proteins brain abundant membrane attached signal protein 1 (Basp1), 3-hydroxybutyrate dehydrogenase type 1 (Bdh1) and guanine nucleotide binding protein (G protein), gamma 12 (Gng12) were regulated by both CIR and CIR+BHD treatments, whereas ADP-ribosylation factor interacting protein 2 **(**Arfip2), guanine nucleotide-binding protein G (i), alpha-1 subunit (Gnai1), guanine nucleotide-binding protein G (i), alpha-2 subunit (Gnai2) and solute carrier family 12, member 5 (Slc12a5) were regulated by both CIR+BHD and CIR+TPA treatments. Only two proteins, S100 calcium binding protein A9 (S100a9) and transthyretin (Ttr) which are involved in calcium signaling and the extracellular region, respectively, were commonly regulated by three different treatments.

### Functional categorization of the regulated proteins

**Fig 3:**
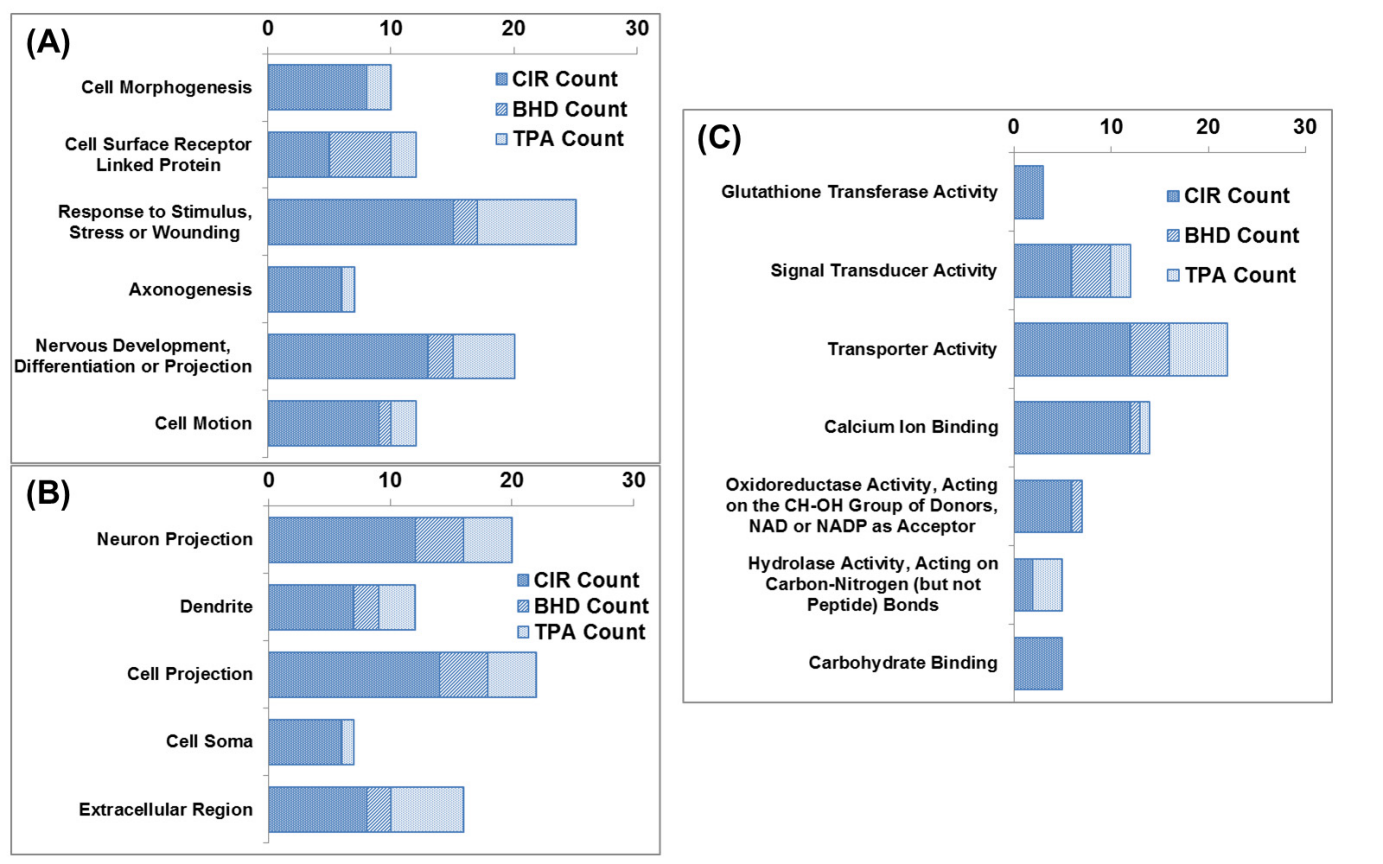
Gene-ontology-based functional categorization of proteins that were significantly changed (fold change > 1.3) by CIR/sham, CIR+TPA/sham and CIR+BHD/sham treatments. (A) Biological Process, (B) Cellular Component, and (C) Molecular Function.

As shown in Fig 3, the proteins that were regulated by CIR, CIR+BHD and CIR+TPA were categorized using a gene ontology (GO) analysis by DAVID [33]. A biological functional categorization (Fig 3A) showed that the CIR mainly regulated the category of proteins which respond to stress, wounding, and stimulus. The CIR+TPA treatment also regulated the proteins which were distributed in the same biological functional categories as those observed for the CIR treatment; however, the number of regulated proteins was ~2-fold lower. In the CIR+BHD group, not only the number in each category was less than that of the CIR group, but also the number of regulated proteins involved in stress or wounding, neuronal development and cell motion was much lower than those observed in the CIR and CIR+TPA groups. In addition, none of the proteins quantified in the CIR+BHD group were involved in axonogenesis and cell morphogenesis, which indicated that the BHD treatment can minimize these two effects after CIR. It is interesting to note that, unlike that which was observed in the CIR and CIR+TPA groups, the majority of the proteins regulated in the CIR+BHD group were in the category of cell surface receptor-linked proteins.

In the cellular component categorization (Fig 3B), the proteins that were regulated by CIR, CIR+TPA and CIR+BHD treatments were in the categories of neuron-related proteins, and most of them were extracellular. Six extracellular proteins, Apolipoprotein E (ApoE), calreticulin (Calr), hyaluronan and proteoglycan link protein 1 (Hapln1), prenylcysteine oxidase 1 like (Pcyox1l), phosphoribosyl pyrophosphate synthetase-associated protein 2 (Psap) and Slc1a3, were regulated only in the CIR group, but not in the CIR+TPA and CIR+BHD groups. However, four extracellular proteins, Alb, C3, Fga and Trf, were regulated in the CIR+TPA group alone. In addition, Ttr was commonly regulated by all three treatments.

A molecular functional categorization (Fig 3C) revealed that the CIR mainly regulated the proteins involved in transporter activity and calcium binding. In the CIR+TPA group, the major regulated proteins were mainly involved in transporter activity, with a minor fraction being involved in calcium binding. Regulated proteins with functions of carbohydrate binding, glutathione transferase and oxidoreductase activities were not observed in the CIR+TPA group. Regarding the CIR+BHD group, the majority of regulated proteins were involved in transport and signal transduction, with a minor fraction being involved in calcium binding. Regulated proteins with functions of carbohydrate binding, glutathione transferase and hydrolase activities were not observed in the CIR+BHD group. It should be noted that the CIR-regulated calcium-binding protein S100a9 was not identified in the groups treated with either BHD or TPA.

### Selected molecular targets of the regulated proteins

To identify the therapeutic effect of BHD or TPA on CIR, it was important to investigate the proteins that exhibited different regulation responses between the CIR and CIR with therapies. The significantly regulated proteins are listed in Table 1. Those proteins were classified into several categories according to their molecular mechanisms. The cellular damaging effect of the CIR was reflected in the induction of proteins involved in apoptosis, BBB breakdown, inflammation/hypoxia, and neurodegeneration. In this study, several self-protective effects induced by the CIR were also observed. Proteins in this category were involved in excitotoxicity, neurogenesis/neuritogenesis, and neuroprotection/brain function. Interestingly, Bdh was the unique BHD-regulated protein, and may be responsible for the major therapeutic effect of BHD.

**Table 1:** Quantitative information of the selected regulated proteins in CIR-induced ischemic stroke mice.

Protein IPI Number	Gene Symbol	Protein Score	Protein Mass	Total Peptide Matches	Unique Peptide Matches	Protein Coverage (%)	Description	CIR / Sham^a^	*p*-value	CIR + BHD / Sham^a^	*p*-value	CIR + TPA / Sham^a^	*p*-value
***Apoptosis***													
IPI00129249	Aplp1	174	77375	16	2	6.3	amyloid beta (A4) precursor-like protein 1	**1.53**	**0.0129**	1.14	0.4049	1.19	0.0928
IPI00125319	Gsk3b	300	54396	16	4	21.2	glycogen synthase kinase 3 beta	**0.57**	**0.0502**	1.01	0.9384	0.96	0.5899
***BBB breakdown***													
IPI00131695	Alb	10170	86122	440	28	36.2	albumin	5.11	0.1484	1.35	0.3900	**2.58**	**0.0039**
IPI00115522	Fga	59	73262	4	2	5.2	fibrinogen alpha chain	1.69	0.7239	1.36	0.1288	**2.04**	**0.0499**
IPI00139788	Trf	687	96369	54	12	13.2	transferrin	3.22	0.1185	1.16	0.1824	**1.75**	**0.0376**
IPI00127560	Ttr	136	18900	6	2	25.9	transthyretin	**3.27**	**0.0231**	**1.58**	**0.0065**	**2.22**	**0.0030**
***CRMPs***													
IPI00122349	Dpysl3	6668	72866	272	22	50.2	dihydropyrimidinase-like 3	1.28	0.0466	1.13	0.2042	**1.31**	**0.0001**
IPI00624192	Dpysl5	1510	71672	114	17	37.2	dihydropyrimidinase-like 5	**1.47**	**0.0133**	1.20	0.0843	**1.30**	**0.0407**
IPI00187545	Plxna4	184	249513	12	4	4.1	plexin A4	**1.37**	**0.0004**	0.87	0.0013	1.07	0.7337
***GABA Receptor***													
IPI00323179	Gdi1	5781	60075	324	23	50.3	guanosine diphosphate (GDP) dissociation inhibitor 1	**1.32**	**0.0018**	1.06	0.4513	1.11	0.0941
IPI00122565	Gdi2	4029	62478	220	17	57.1	guanosine diphosphate (GDP) dissociation inhibitor 2	**1.34**	**0.0187**	1.06	0.3063	1.12	0.1553
IPI00227838	Gng12	107	10214	17	3	47.2	guanine nucleotide binding protein (G protein), gamma 12	**0.77**	**0.0101**	**0.72**	0.0168	0.85	0.2599
IPI00467152	Gnai1	2285	50225	102	4	35.9	guanine nucleotide binding protein (G protein), alpha inhibiting 1	0.67	0.2591	**0.77**	**0.0134**	**0.71**	**0.0000**
IPI00652902	Gnai2	2202	49441	103	2	28.7	guanine nucleotide binding protein (G protein), alpha inhibiting 2	0.65	0.2476	**0.77**	**0.0148**	**0.70**	**0.0000**
***Glutamate excitotoxicity***													
IPI00114279	Slc1a3	3948	68544	131	7	14.7	solute carrier family 1 (glial high affinity glutamate transporter), member 3	**1.38**	**0.0017**	1.02	0.8508	1.04	0.0660
IPI00230289	Slc1a2	5290	72183	317	18	26.0	solute carrier family 1 (glial high affinity glutamate transporter), member 2	0.71	0.0634	0.66	0.1310	**0.58**	**0.0179**
IPI00125397	Slc30a3	56	43990	6	3	4.6	solute carrier family 30 (zinc transporter), member 3	1.08	0.2297	0.71	0.0528	**0.63**	**0.0444**
IPI00314749	Slc4a4	853	146782	52	11	13.4	solute carrier family 4 (anion exchanger), member 4	**0.59**	**0.0186**	0.74	0.2734	0.76	0.3535
IPI00465769	Slc12a5	1923	137784	133	19	19.8	solute carrier family 12, member 5	0.53	0.1041	**0.74**	**0.0078**	**0.66**	**0.0163**
IPI00553387	Grm5	44	148445	2	2	2.2	glutamate receptor, metabotropic 5	**0.67**	**0.0445**	0.87	0.4601	0.59	0.2529
***Inflammation and hypoxia***													
IPI00323624	C3	95	224415	13	4	3.7	complement component 3	2.44	0.1303	0.82	0.5721	**1.31**	**0.0054**
IPI00126405	Mog	524	31008	23	3	7.7	myelin oligodendrocyte glycoprotein	**1.40**	**0.0053**	1.07	0.3536	1.12	0.4381
IPI00222556	S100a9	87	16829	4	2	12.4	S100 calcium binding protein A9 (calgranulin B)	**2.68**	**0.0218**	**1.34**	**0.0152**	**1.53**	**0.0448**
IPI00230212	Gstm1	2428	31825	119	7	48.6	glutathione S-transferase, mu 1	**0.72**	**0.0452**	1.00	0.9866	0.98	0.6764
IPI00114380	Gstm5	486	33023	43	7	28.1	glutathione S-transferase, mu 5	**0.64**	**0.0086**	1.03	0.0841	1.05	0.3596
IPI00555023	Gstp1	2628	27687	77	6	35.2	glutathione S-transferase, pi 1	**0.55**	**0.0069**	1.16	0.4272	1.18	0.0325
***Metabolism***													
IPI00330754	Bdh1	192	45837	22	6	9.9	3-hydroxybutyrate dehydrogenase, type 1	**0.57**	**0.0489**	**1.34**	**0.0116**	1.31	0.1144
***Neurodegernation***													
IPI00230151	Mag	889	69222	74	6	11.7	myelin-associated glycoprotein	**1.46**	**0.0412**	1.31	0.4231	1.33	0.5003
***Neurogenesis/Neuritogenesis***													
IPI00380436	Actn1	616	119937	27	7	10.1	actinin, alpha 1	**4.04**	**0.0442**	1.25	0.0157	1.70	0.1345
IPI00119870	Ctnna2	822	127158	31	4	9.1	catenin (cadherin associated protein), alpha 2	**1.40**	**0.0122**	1.03	0.6757	1.13	0.0743
IPI00320420	Clu	419	61255	23	3	10.0	clusterin	**1.63**	**0.0039**	1.07	0.0352	**1.35**	**0.0105**
IPI00128973	Gap43	1901	33141	106	9	56.8	growth associated protein 43	**2.20**	**0.0252**	1.08	0.2446	1.08	0.0049
IPI00117042	Gfap	1743	56304	122	17	45.6	glial fibrillary acidic protein	3.55	0.0626	1.21	0.0615	**1.35**	**0.0286**
IPI00329927	Nfasc	948	155569	46	12	14.4	neurofascin	**1.41**	**0.0009**	0.97	0.1281	0.97	0.6982
IPI00338880	Nrcam	892	154088	37	9	11.3	neuron-glia-CAM-related cell adhesion molecule	**1.32**	**0.0199**	1.03	0.3844	1.01	0.8675
IPI00230050	Nrxn1	193	191808	9	4	5.2	neurexin I	**1.37**	**0.0234**	1.06	0.7173	1.08	0.3370
IPI00263013	Plp1	1957	34656	162	8	22.4	proteolipid protein (myelin) 1	**2.04**	**0.0275**	1.05	0.7693	1.23	0.6323
IPI00129519	Basp1	5046	30896	179	12	75.7	brain abundant, membrane attached signal protein 1	**0.67**	**0.0121**	**0.70**	**0.0000**	0.77	0.2670
IPI00110990	Dusp3	195	23381	12	2	13.5	dual specificity phosphatase 3 (vaccinia virus phosphatase VH1-related)	**0.54**	**0.0411**	1.02	0.5916	1.06	0.5522
***Neuroprotection/Brain function***													
IPI00221845	Atcay	59	46245	9	3	9.4	ataxia, cerebellar, Cayman type homolog (human)	**1.33**	**0.0158**	1.14	0.1953	1.05	0.3401
IPI00453537	Cadm2	926	52942	46	6	19.3	cell adhesion molecule 2	**1.37**	**0.0230**	0.95	0.0872	1.03	0.6947
IPI00312058	Cat	246	69996	7	3	6.6	catalase	**1.37**	**0.0074**	1.20	0.0955	**1.36**	**0.0040**
IPI00123058	Cntn1	3529	131346	217	31	30.5	contactin 1	**1.42**	**0.0120**	0.81	0.0374	0.82	0.2230
IPI00109727	Thy1	1886	21295	90	7	35.8	thymus cell antigen 1, theta	0.39	0.1524	**0.57**	**0.0308**	0.56	0.0581
IPI00122069	Prkcc	895	91183	63	12	19.5	protein kinase C, gamma	**0.49**	**0.0340**	0.86	0.4856	0.72	0.3239
IPI00130419	Prkce	335	101950	27	6	10.3	protein kinase C, epsilon	**0.59**	**0.0092**	0.88	0.4316	0.87	0.2304
IPI00314191	Cbr1	407	37240	32	5	43.0	carbonyl reductase 1	**0.32**	**0.0004**	0.97	0.4248	0.93	0.2773
IPI00222430	Dbi	503	20436	31	3	25.2	diazepam binding inhibitor	1.15	0.1776	1.25	0.0481	**1.31**	**0.0143**
IPI00408909	Mtap1a	2119	360685	118	20	10.3	microtubule-associated protein 1 A	**0.65**	**0.0000**	0.99	0.7619	0.86	0.0002
IPI00406741	Mtap4	62	120427	5	3	5.7	microtubule-associated protein 4	**0.56**	**0.0003**	0.89	0.2505	0.92	0.1802
IPI00114939	Nptxr; Cbx6; Cbx6-Nptxr	681	57883	17	2	4.5	neuronal pentraxin receptor; chromobox homolog 6; Cbx6-Nptxr readthrough transcripts	**1.41**	**0.0248**	1.01	0.8927	0.92	0.5202
IPI00463761	Syn3	71	72615	15	2	11.9	synapsin III	**0.48**	**0.0225**	0.98	0.7368	0.89	0.1896

^a^Geometric Mean of Three Biological Replicates. They were classified according to their participation in the key molecular events of ischemic stroke pathophysiology.

The ratios with significant p-value (<0.05) are shown in bold.

### Validation of regulated proteins

**Fig 4:**
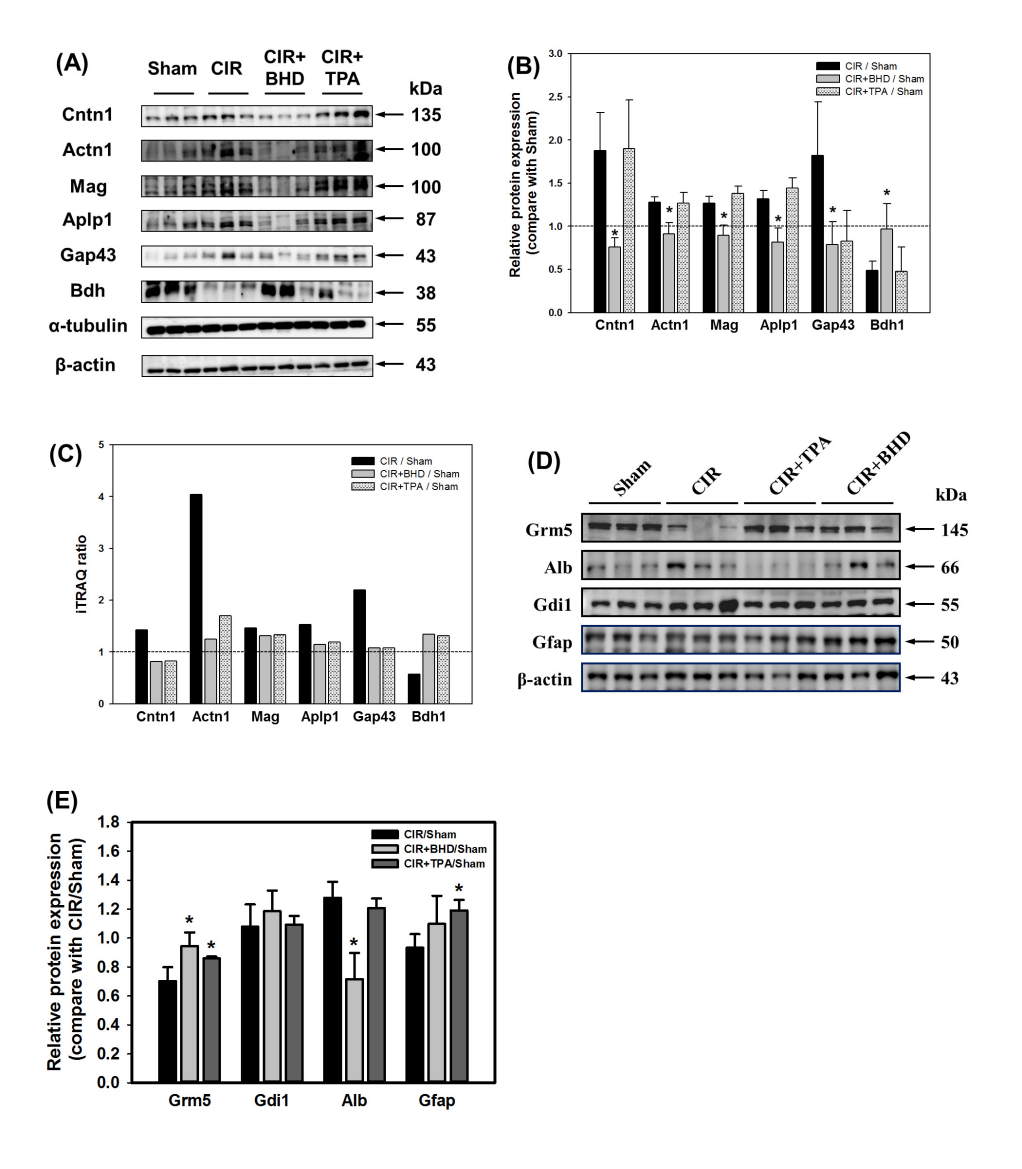
Validation of the selected proteins by western blotting. (A) The levels of upregulated proteins (Cntn1, Actn1, Mag, Aplp1 and Gap43) and downregulated protein (Bdh) affected by CIR were recovered to the basal situation in the BHD-treated group (compared with the sham group). (B) Quantitative analysis of the selected proteins mentioned above. The values represent the ratio of the treatment group compared with the sham group, using α-tubulin as a loading reference. (C) The histogram indicates the ratio of the proteins selected in the iTRAQ experiment. (D) The levels of upregulated proteins (Alb and Gdi1) and downregulated proteins (Grm5 and Gfap) affected by CIR were reversed in the BHD-treated group (compared with the sham group). (E) Quantitative analysis of the selected proteins mentioned above. The values represent the ratio of the treatment group compared with the sham group, using β-actin as a loading reference. Data are presented as the mean ± S.D. Each protein expression with triplicates was statistically analyzed using Student *t*-test. *p < 0.05 compared with CIR/Sham.

After the database search and classification of proteins, western blotting of the selected proteins was performed to verify the iTRAQ results. As shown in Fig 4, ten selected proteins (contactin 1 (Cntn1), actinin, alpha 1 (Actn1), myelin-associated glycoprotein (Mag), amyloid beta precursor-like protein 1 (Aplp1), growth associated protein 43 (Gap43) and Bdh), Grm5, Alb, Gdi1, and Gfap were further evaluated by western blotting, and α-tubulin as well as β-actin were used as internal controls. Results showed that both iTRAQ experiment and Western blotting with similar trend (Table 1 and Fig 4A–4E).

### Evaluation of protein phosphorylation

**Fig 5:**
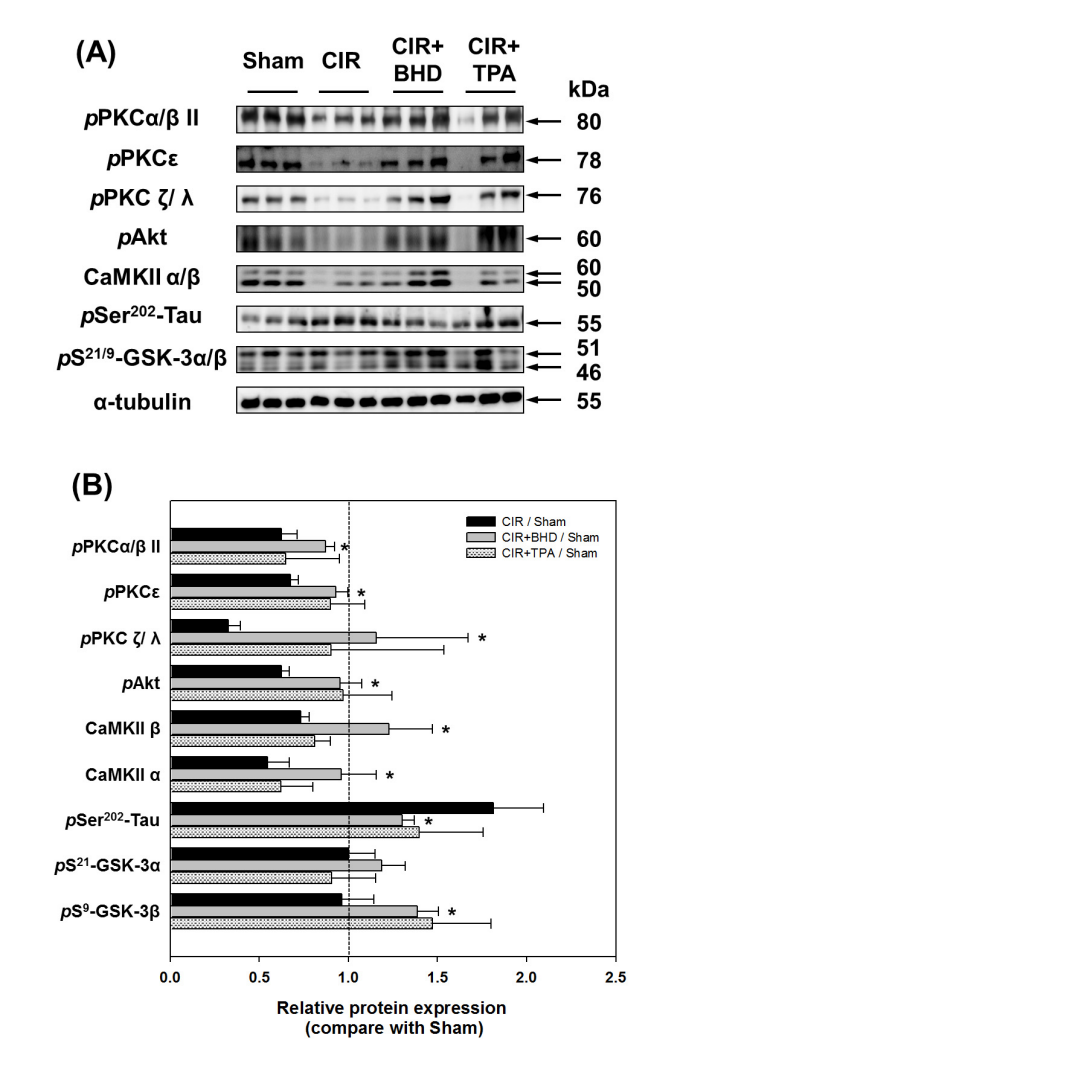
Neuroprotective effects of BHD on the GSK-3 pathway. (A) BHD treatment significantly increased the phosphorylation level of PKCs, Akt, CaMKII and GSK-3; thus, inhibiting Tau phosphorylation yielded neuroprotective activity. (B) Quantitative analysis of the phosphorylated proteins was mentioned above. The values represent the ratio of the treatment group compared with the sham group, using α-tubulin as a loading reference. Data are presented as the mean ± S.D. Each protein expression with triplicates was statistically analyzed using Student *t*-test. *p < 0.05 compared with CIR/Sham.

The iTRAQ experiment led to the identification of a series of novel proteins including structural and extracellular proteins. However, some protein kinases (such as GSK-3) were also observed, albeit with less statistical significance. The evaluation of the levels of phosphorylation of proteins is important for unraveling their functional effects. Related proteins were selected to estimate the levels of phosphorylation by western blotting. After the BHD treatment, the phosphorylation of GSK-3α/β was dramatically increased when compared with those observed in the CIR group (Fig 5). In addition, the phosphorylation of the GSK-3 upstream kinases protein kinase C (PKC) and Akt was significantly increased in the BHD-treated group. Moreover, the level of phosphorylation of Tau on Ser-202 by GSK-3, which was increased in the CIR group, was significantly decreased in the BHD-treated group. These results were consistent with the findings of GSK-3α/β phosphorylation, and showed the inhibitory effects of GSK-3 activity. In addition, the expression of CaMKIIα/β was restored in the BHD-treated group when compared with the CIR group. However, the levels of phosphorylated proteins in the TPA-treated group were similar to those observed in the BHD-treated group, albeit with lower significance.

### BHD preserved the BBB by increasing Occludin and CaMKII and reducing apoptosis in infarct areas

**Fig 6:**
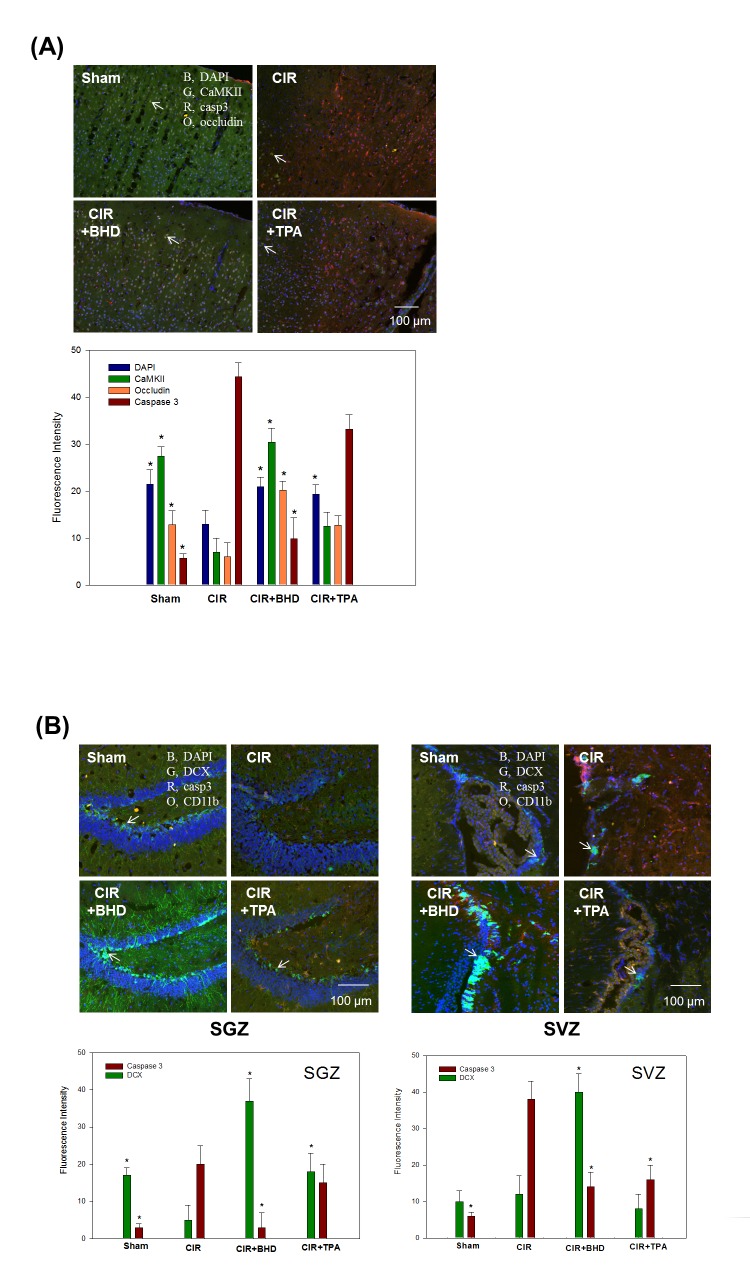
Protective effects of BHD against ischemic stroke-injured mice, as revealed by immunohistochemical staining. (A) Brain slices were taken 1.5–1.7 mm caudal to the bregma 24 h after stroke. Blood–brain barrier (BBB) integrity was assessed by the staining of occludin (O, orange); apoptosis was assessed by caspase 3 staining (R, red); preserved areas were assessed by calcium/calmodulin-dependent protein kinase II (CaMKII) staining (G, green); DAPI (blue, a marker of nuclei). (B) Neurogenesis was examined on day 3 after stroke. Arrows indicate staining of doublecortin (DCX) (G, green), a marker of neuronal stem cells; and CD11b (O, orange), a marker of inflammatory cells. Animal groups included sham, vehicle-treated animals (stroke, CIR), BHD-treated animals (CIR+BHD; 1.0 g/kg, p.o., twice daily), and TPA-treated animals (CIR+TPA; 10 mg/kg, i.v.), with treatment administered 2 h after ischemic stroke. At least three independent experiments were performed in this study.

The proteomics study showed that the levels of Alb, Fga and Trf in the BHD group were similar to those of the sham group (Table 1, *BBB breakdown* sector). We hypothesized that BHD prevented BBB breakdown. Moreover, the upregulation of CaMKII implied that BHD prevented neuronal death. Therefore, brain slices were collected at 24 h after the CIR to perform an IHC study. The results showed that the CIR-induced brain injury was closely associated with the facilitated BBB breakdown (loss of occludin staining), neuronal death (loss of CaMKII staining), and apoptotic cell death (increased caspase 3 staining) (Fig 6A; Quadrant I). BHD significantly preserved the BBB and neurons, suppressed apoptotic cell death, and was more effective than TPA (Fig 6A; Quadrants III and IV, respectively). These results indicate that BHD is capable of reducing the CIR-induced disruption of the BBB integrity and of enhancing neuronal cell survival to a greater extent than the TPA treatment.

### BHD promoted endogenous neurogenesis within the subgranular zone (SGZ) and the subventricular zone (SVZ) of CIR mice

Potential neural stem/progenitor cells are present in various brain regions, including the SGZ of the hippocampal dentate gyrus and the SVZ of the lateral ventricle, thus allowing the restoration of the brain function by these cells after CIR [34]. To explore whether endogenous neurogenesis was promoted by BHD and/or TPA treatments at day 3 after the ischemic stroke, we used doublecortin (DCX, a marker of newborn neuroblasts) to assess the endogenous neurogenesis. In this study, the CIR mice exhibited relatively low staining for DCX within the SGZ and SVZ compared with the sham mice (Fig 6B; Quadrant I). Moreover, caspase 3 (apoptosis marker) staining was significantly increased in the CIR group, which denoted the presence of apoptotic neuronal death. CIR mice treated with BHD, but not with TPA, exhibited significant upregulation of DCX (compared with the CIR group), which was accompanied by the downregulation of CD11b (inflammatory marker) and caspase 3 (Fig 6B; Quadrant III).

## Discussion

Previously, we used genomics to assess the effect of BHD on genome-wide expression profiling *via* an mRNA microarray study [18]. Furthermore, an NMR-based metabolomics study was performed to clarify the metabolite-based mechanism of BHD in stroke mice [19]. In this proteomics study, we demonstrated that BHD treatment (1.0 g/kg, p.o., twice daily) significantly improved motor function in CIR mice by preserving the integrity of the BBB and suppressing excitotoxicity; thus indicated a novel neuroprotective effect of BHD in the stroke mice. Moreover, BHD-enhanced neurogenesis and mediated metabolism were confirmed, which is consistent with the results of our previous study [18, 19]. The iTRAQ-based proteomics study provided an overall effective tool to evaluate the effects of multiple-target TCM and generated a novel holistic perspective on the effects of BHD. In the present study, the administered dose of TPA (10 mg/kg, i.v.) was according to the suggestion by the National Institute of Neurological Disorders and Stroke [35]. We found that the TPA treatment prevented mouse death by only 10% and yielded a lesser motor function recovery. These results imply the limited applicability of TPA.

### BHD treatment significantly preserved BBB integrity in CIR-induced ischemic stroke mice

Loss of the BBB function is one of the etiologic components of stroke. The breakdown of the BBB may cause a leakage of brain-specific proteins to the circulation, which might potentially represent serum-specific biomarkers for predicting therapeutic outcome and stroke progression [36]. The present study showed that S100a9, a brain-specific protein, was upregulated in the CIR mice, and Gfap showed an increasing trend. Furthermore, three BBB-damage-related proteins (Alb, Fga, and Trf) were recovered from the CIR-induced brain damage after the BHD treatment (Table 1, *BBB breakdown sector*). Alb leakage is a BBB permeability marker which is used to evaluate ischemic stroke. The CIR-induced brain damage resulted in increased Alb level, probably due to its infiltration from the blood plasma as a result of breakage of the BBB [37]. Fga is not only a marker of the BBB breakdown, but also plays a causative role in neurological disease as a potent inducer of inflammation and an inhibitor of neurite outgrowth [38]. Fga deposition has also been reported in the MCAO mouse brain [39]. In this study, the BHD treatment significantly reduced the deposition of Fga, which may preserve BBB integrity and improve neurogenesis. In addition, Trf, a marker of neuroinflammation, is positively associated with the BBB breakdown [40]. Datta et al. reported that Trf is upregulated in the MCAO rat brain and may be involved in deregulated iron homeostasis [41]. Our proteomics study showed that the BHD treatment, but not the TPA treatment, recovered Alb, Fga, and Trf expressions to normal levels, which was consistent with the IHC data (Fig 6A). These results indicate that the BHD treatment significantly preserved the BBB integrity after the CIR-induced ischemic stroke. Although TPA is the only FDA-approved treatment for ischemic stroke, Jin et al., reported that the TPA treatment worsens the disruption of the BBB [42]. In this study, the upregulation of Alb, Fga, and Trf in the TPA-treated group also suggests that the TPA treatment may further aggravate the BBB damage in the CIR-induced brain injury. Western blotting also showed Alb expression in BHD treatment was significantly decreased but not in TPA group (Fig 4D and 4E).

### BHD treatment modulated excitotoxicity in ischemic stroke

γ-Aminobutyric acid (GABA) is the main inhibitory neurotransmitter in the brain and modulates the inhibitory–excitatory balance for brain function. In this study, the majority of regulated proteins in the CIR+BHD group were in the category of “biological function- cell surface receptor-linked proteins” in the GO analysis (Fig 3A). The selected proteins that exhibited significant changes, including Gnai1, Gnai2, GDP dissociation inhibitor alpha (Gdi1) and GDP dissociation inhibitor beta (Gdi2), were involved in GABA_B_ receptor activation (Table 1, *GABA Receptor* sector). The Rho Gdi negatively regulates the Rho GTPase activity, which results in neuronal cell death [43]. Our results (Table 1 and Fig 4) showed that the BHD treatment restored both Gdi1 and Gdi2 protein levels thereby reducing the cell death, which was consistent with our previous study [19]. Despite the fact that the α subunits of G protein were not identified in our study, the levels of inhibitory Gnai1 and Gnai2 were decreased in the BHD-treated group. This result implied that the neurotransmitter transduction was activated by the BHD treatment. Although the expression of the GABA receptor was enhanced in both the BHD- and TPA-treated groups, the outcome of motor function was different. A failed clinical trial of a GABA agonist showed that TPA rescued GABA receptor-related proteins, albeit without a therapeutic effect on the therapy of stroke [44]. Therefore, increasing a GABA receptor signaling alone is not sufficient to overcome an ischemia-induced stroke.

Glutamate is the major excitatory synaptic neurotransmitter in CNS. Glutamate excitotoxicity is the primary cause of acute neuronal death and initiates apoptosis after ischemia. According to a GO analysis of biological process, the “response to stimulus, stress, or wounding,” category was significantly changed after the CIR-induced ischemic stroke. The significantly changed proteins selected, including glutamate receptor metabotropic 5 (Grm5 or metabotropic glutamate receptor 5 (mGluR5)), Slc1a2, and Slc1a3, were involved in glutamate excitotoxicity (Table 1, *Glutamate excitotoxicity* sector). Glutamate activates either ionotropic glutamate receptors (GluRs) or G-protein-coupled metabotropic receptors (mGluRs). The Grm5 (the name of the gene that encodes mGluR5), which is a subtype of group I mGluR, is highly expressed in neurons and astrocytes, is especially localized in the postsynaptic density area, and plays a role in modulating synaptic plasticity and neuronal excitation [45]. In this study, we found that BHD treatment reversed CIR-induced Grm5 expression but not TPA (Table 1 and Fig 4D and 4E). Li et al. reported that inhibition of mGluR5 is beneficial to reduce brain damage and improve long-term potentiation [46]. Controversially, Nochi et al., showed that mGluR signaling is responsible for promoting the proliferation of neural stem cells after MCAO [47]. However, both an agonist and an antagonist of Grm5 showed neuroprotective effects in MCAO mice by ameliorating neuronal death and improving functional recovery, respectively [48]. Therefore, the downregulation of Grm5 in a CIR-induced stroke indicated a self-protective mechanism of the brain, albeit with less neurogenesis, which might result in a poorer motor function.

Excitatory amino-acid transporters (EAATs) are essential for maintaining normal extracellular levels of glutamate, and five distinct EAATs have been identified in the brain. Among these EAATs, the glial glutamate/aspartate transporter (GLAST) and the glial glutamate transporter-1 (GLT-1) are the major transporter which is localized primarily in astrocytes, and prevent chronic glutamate neurotoxicity [49]. Within many brain regions, GLT-1, which is the dominant glutamate transporter, plays an essential role in removing glutamate from the extracellular space and maintaining glutamate below neurotoxic levels in the brain. Our results showed that Slc1a2 (the name of the gene that encodes GLT-1), displayed a downregulated trend after the CIR-induced ischemic stroke, thus pinpointing the induction of excitotoxicity in the brain. Although Slc1a3 (the name of the gene that encodes GLAST) was upregulated in CIR mouse brain, which may be associated with the improved neurological outcome observed after MCAO [50], GLT-1 plays a main regulatory role and contributes to more than 90% of the glutamate transport in the brain. Taken together, these results showed that glutamate excitotoxicity was observed in the CIR mouse brain. The normal level of Slc1a3 in the CIR+BHD group may indicate that the BHD treatment potentially preserves the normal effect on glutamate release (Table 1 and Fig 4). This hypothesis was also supported by the lower cell death in IHC imaging. In summary, the normal function of mGluR and EAATs in the BHD-treated mice yielded lower excitotoxicity and neuroprotective activity.

### The irreversible proteins after CIR-induced ischemic stroke

After the BHD or TPA treatment, the levels of most of the proteins were restored to the basal level as compared to the sham group. These results indicate that brain damage in the BHD- or TPA-treated mice was less severe than that observed in the CIR mice. However, the levels of some proteins such as S100a9 and Ttr remained irreversible after the BHD or TPA treatment. S100a9, also known as myeloid-related protein 14 (MRP-14) or calgranulin-B, is a calcium-binding protein that is found mainly in glial cells. Zigeler et al. demonstrated that the level of S100a9 was increased in the mouse brain after focal cerebral ischemia. S100a9 was characterized as an endogenous TLR-4 agonist and may contribute to neuroinflammation and the progression of ischemic damage [51]. In addition, Ttr, which is primarily localized in the choroid plexus, may be a biomarker of blood-to-cerebrospinal barrier disruption [52]. In this study, the significant upregulation of S100a9 and Ttr in the CIR mouse brain indicated the successful induction of a stroke model and the reliability of the proteomics study. Although the levels of S100a9 and Ttr were significantly higher when compared with the sham group, a decreased trend was observed in the BHD-treated group. S100a9 expression was significantly downregulated in the BHD-treated mouse brain, as was also observed in our previous genomics study [18], which indicated that both the BBB damage and inflammation were ameliorated by BHD. In BHD-treated mice, the CIR-induced downregulation of the Basp1 and Gng12, could not be recovered. Gap43 and Basp1 are important structural proteins in neurogenesis included in neurite outgrowth [41, 53]. Our study showed that the upregulation of Gap43 after the CIR-induced ischemic stroke may be caused by a self-repairing mechanism in the brain. According to the recovery of Gap43 expression demonstrated in the proteomics study (Table 1, *Neurogenesis* sector) and western blotting experiment (Fig 4A), the brain damage was less severe in the BHD-treated mice. Despite the fact that the Basp1 level in the CIR+BHD group was still lower than that observed in the sham group, Basp1 was unable to replace the neurogenesis role of Gap43 on neural cell adhesion molecule (NCAM)-mediated neurite outgrowth [53]. In addition, in this study, neither Ncam1 nor Ncam2 was significantly changed in any one of the groups (data not shown). It should be noted that Bdh1 was significantly increased in the BHD-treated mice, but dramatically decreased in the CIR group (Table 1 and Fig 4A–4C). Although the relationship between Bdh and stroke has not, as yet, received very much attention, Kim et al., reported that the upregulation of Bdh after ischemic heart damage may play a cellular-protection role against ischemia [54]. The enhancement of Bdh1 may imply an energy transfer by ketone bodies, which is consistent with our previous metabolomics study showing that 3-hydroxybutyrate was significantly downregulated in the BHD-treated mouse brain [19]. This result indicated that BHD may mediate the energy metabolism and prevent ketosis in the brain. However, the irreversible proteins in the TPA group included Clu, Dpysl5, and Cat. These proteins were significantly increased after the CIR-induced stroke, and remained at a higher level in the TPA-treated group. The upregulation of Clu has been reported in the MCAO mouse brain in a neuroprotective role by influencing the long-term structural remodeling process [55]. Dpysl5 is a member of the collapsin response mediator family of proteins (CRMPs) and is an important brain-specific protein with distinct functions in the modulation of growth cone collapse and axonal guidance during brain development [56]. In addition, Dpysl3 (the name of the gene that encodes CRMP4) showed an upregulated trend in the CIR-induced mouse brain (Table 1, *CRMPs* sector). Neurodegenerative disorders are associated with CRMPs induction, such as the high level of expression of CRMP4 observed in the rat brain after transient brain ischemia, which results in neurogenesis [57]. Moreover, Cat prevents H_2_O_2_ toxicity by boosting H_2_O_2_ enzymatic degradation to H_2_O and O_2_ thus suppressing oxidative stress [58]. Taken together, our results suggest that the upregulation of Clu, Dpysl5, and Cat is associated with a self-compensation mechanism against ischemic stroke.

### BHD showed the neuroprotective effects may through the inactivation of GSK-3 activity

GSK-3 is a cytoplasmic serine/threonine kinase, the dysfunction of which may be linked to the pathophysiology of numerous disorders including various neurodegenerative diseases. In a clinical trial, selective GSK-3 inhibition by lithium was shown to be protective against cerebral ischemic [59]. Although we observed a lower expression of GSK-3β in the CIR group in the iTRAQ experiment, the activity and function of GSK-3β cannot be evaluated based on total protein expression. In particular, reversible phosphorylation catalyzed by kinases and phosphatases is a key step in the cellular signaling that initiates various cellular functions such as growth, metabolism, and differentiation. GSK-3 activity is inhibited *via* the phosphorylation of its N-terminal serine by several protein kinases such as Akt, PKC and CaMKII [60–62]. The Akt pathway contributes to neuronal survival after stroke by causing GSK-3 phosphorylation and leading to GSK-3 inactivation [60]. Our results showed that Akt phosphorylation was increased, which paralleled the enhancement of GSK-3 phosphorylation, in either the BHD- or TPA-treated mouse brain (Fig 5A). We suggest that the BHD and TPA treatments enhance neuronal survival after stroke. PKC has a potent neuroprotective activity, including the regulation of cell apoptosis and survival in neurodegenerative diseases [61, 63]. Our study showed that both BHD and TPA treatments activated PKCs, resulting in GSK-3 inactivation and the promotion of neurogenesis. This may be in agreement with a recent report showing that a PKC activator improved the survival rate and functional outcome in an MCAO rat model [64]. The CaMKIIα/β is expressed most abundantly in neurons and is involved in the regulation of many neuronal functions. CaMKII functions as a direct upstream kinase of GSK-3 and mediates depolarization-dependent survival in neurons by inducing the inhibitory phosphorylation of GSK-3 [61]. Moreover, Tau, which is also known as microtubule-associated protein tau (Mapt), is abundant in neurons and plays a critical role in neurodegenerative diseases. The hyperphosphorylation of Ser202 of Tau induced by GSK-3 contributes to ischemic neuronal injury [17]. In this study, the lower level of GSK-3 phosphorylation and the higher level of Tau phosphorylation observed in the CIR mice indicated the presence of ischemia-induced neuronal death. Taken together, our findings demonstrated that BHD attenuated cerebral ischemic/reperfusion-induced Tau phosphorylation, most likely *via* inhibition of the activity of GSK-3 by enhancing Akt, PKC, and CaMKII expression.

Although the repair capacity of the CNS is limited, it has been proven to have the potential to repair brain injuries, such as stroke, by activating endogenous neurogenesis [65]. In this proteomics study, the neurogenesis-related proteins, Actn1, Gap43, Gfap, and neurofascin (Nfasc) were upregulated after the CIR-induced stroke (Table 1, *Neurogenesis* sector). Nevertheless, GSK-3 and Tau activity remained higher in the CIR group, which implies that neurogenesis is impaired in the CIR mice. In our previous genomics study, the endogenous neurogenesis gene, *DCX*, was significantly upregulated after the BHD treatment [18]. However, DCX has not been detected in this proteomics study, which may be explained by differences in sampling time or in the relatively small amount of stem cells expressed in specific areas. After the BHD treatment, the significant upregulation of DCX (Fig 6B) was consistent with the increased GSK-3 phosphorylation and the decreased Tau dephosphorylation observed by western blotting (Fig 5A). These results explained the increase in the normal motor function detected in the CIR+BHD group. These effects of BHD provide a novel therapeutic perspective associated with the enhancement of endogenous neurogenesis.

## Conclusion

**Fig 7:**
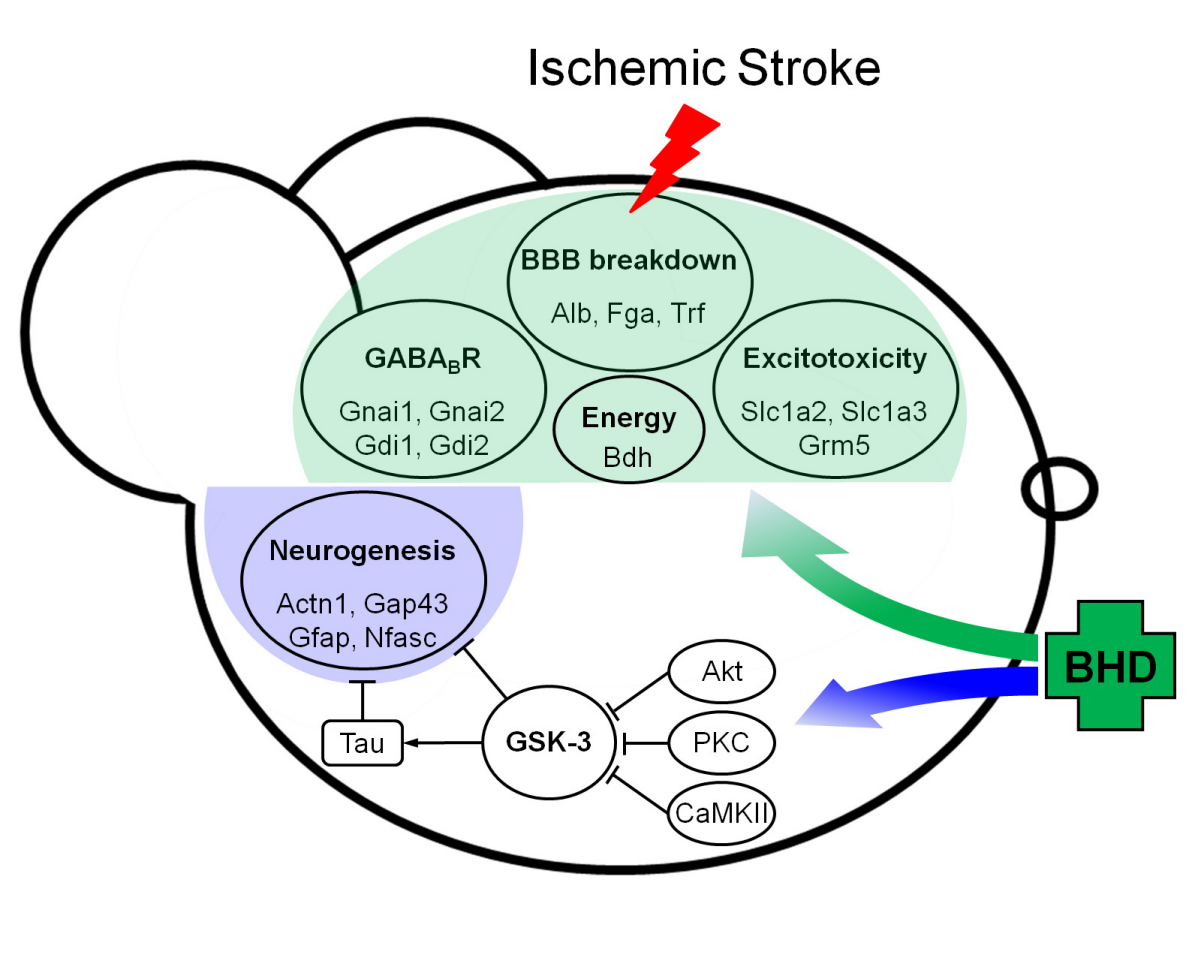
Summary of the overall findings of the iTRAQ-based proteomics analysis. BHD treatment significantly preserved the integrity of the BBB, suppressed glutamate excitotoxicity, and improved the energy metabolism. Mechanistically, BHD enhanced kinase activity (Akt, PKC, and CaMKII), thereby inhibiting GSK-3 and Tau activity, which suggests a neuroprotective effect.

iTRAQ-based proteomics technology was used to unravel the mechanism underlying the effects of BHD in the CIR-induced stroke mice and to provide clearer and more scientific evidence of the applications of Chinese medicine. As shown in Fig 7, the BHD treatment effectively prevented BBB breakdown, suppressed excitotoxicity-mediated cell death, prolonged survivability, and promoted locomotor function after stroke. Specifically, the BHD treatment exerted neuroprotective effects likely *via* the inactivation of GSK-3 and Tau activity by inhibitory modulation of phosphorylation. The phosphoproteomics study may be a recommended strategy in any future work. However, TPA induced BBB breakdown, which may now be considered as a major side effect of stroke treatment. To our knowledge, this was the first proteomics study that evaluated the therapeutic efficacy of a Chinese herbal prescription. Finally, this study has provided scientific-based evidence that supports BHD as a complementary therapy in stroke.

## Supporting Information

S1 Fig HPLC profile of BHD. (TIF)

S2 Fig Scheme of the present experiments. (TIF)

S3 Fig The chromatogram of SCX. (TIF)

S1 Table Antibodies used in this experiment. (DOC)

S2 Table List of Quantified and Identified Proteins in Mouse Brain. (XLSX)

S3 Table Functional Categorization of Total Identified Proteins and the Proteins Only Regulated by CIR, CIR+BHD and CIR+TPA, Respectively. (XLSX)

## Abbreviations

Actn1: α-actinin
Arfip2: ADP-ribosylation factor interacting protein 2
Alb: albumin
Aplp1: amyloid beta precursor-like protein 1
Bdh1: 3-hydroxybutyrate dehydrogenase type 1
BBB: blood–brain barrier
Basp1: brain abundant membrane attached signal protein 1
BHD: Buyang Huanwu Decoction
CaMKII: Ca^2+^/calmodulin-dependent protein kinase II
Cat: catalase
Calr: calreticulin
CIR: cerebral ischemia/reperfusion
Clu: clusterin
C3: complement component 3
Cntn1: contactin
CRMPs: collapsin response mediator proteins
DAPI: 4' 6-diamidino-2-phenylindole
Dbi: diazepam binding inhibitor
Dpysl5: dihydropyrimidinase-like 5
DCX: doublecortin
Dbn1: drebrin 1
EAATs: excitatory amino-acid transporters
Erp44: endoplasmic reticulum resident protein 44
Eprs: glutamyl-prolyl-tRNA synthetase
Fga: fibrinogen alpha chain
GABA: γ-aminobutyric acid
Gabrb3: gamma-aminobutyric acid receptor subunit beta-3
Gdi1: GDP dissociation inhibitor alpha
Gdi2: GDP dissociation inhibitor beta
GO: gene ontology
Gfap: glial fibrillary acidic protein
GLT-1: glial glutamate transporter-1
GLAST: glial glutamate/aspartate transporter
Grm5: glutamate receptor metabotropic 5
GluRs: glutamate receptors
Gng12: guanine nucleotide binding protein (G protein), gamma 12
GSK-3: glycogen synthase kinase-3
Gap43: growth-associated protein 43
Gnai: guanine nucleotide binding protein, alpha inhibiting
Hapln1: hyaluronan and proteoglycan link protein 1
Hba-a1: hemoglobin alpha, adult chain 1
IHC: immunohistochemical
mGluRs: metabotropic glutamate receptors
MMTS: methyl methanethiosulfonate
Mapt: microtubule-associated protein tau
MCAO: middle cerebral artery occlusion
Napa: N-ethylmaleimide-sensitive factor attachment protein, alpha
NCAM: neural cell adhesion molecule
Nfasc: neurofascin
NRICM: National Research Institute of Chinese Medicine
Pcyox1l: prenylcysteine oxidase 1 like
Psap: phosphoribosyl pyrophosphate synthetase-associated protein 2
PI3K: phosphoinositide 3-kinase
PET: positron-emission tomography
Psmd: 26S proteasome non-ATPase regulatory
PKC: protein kinase C
S100a9: S100 calcium binding protein A9
Slc1a2: solute carrier family 1, member 2
Slc1a3: solute carrier family 1, member 3
SGZ: subgranular zone
SVZ: subventricular zone
TPA: tissue plasminogen activator
TCM: traditional Chinese medicine
Thy1: thymus cell antigen 1, theta
Them6: RIKEN cDNA 4930572J05 gene
Trf: transferring
Ttr: transthyretin
Tnpo2: transportin 2
TEABC: triethylammonium bicarbonate
TCEP: tris (2-carboxyethyl) phosphine hydrochloride

## References

[1] World Health Organization. "The 10 leading causes of death in the world, 2000 and 2012": Available: http://www.who.int/mediacentre/factsheets/fs310/en/.

[2] Lo EH, Dalkara T, Moskowitz MA. Mechanisms, challenges and opportunities in stroke. Nat Rev Neurosci. 2003;4:399–415. 12728267 10.1038/nrn1106

[3] Fernandez-Lopez D, Faustino J, Daneman R, Zhou L, Lee SY, Derugin N, et al Blood-brain barrier permeability is increased after acute adult stroke but not neonatal stroke in the rat. J Neurosci. 2012;32:9588–9600. 10.1523/JNEUROSCI.5977-11.2012 22787045 PMC3539825

[4] Mangi AA, Noiseux N, Kong D, He H, Rezvani M, Ingwall JS, et al Mesenchymal stem cells modified with Akt prevent remodeling and restore performance of infarcted hearts. Nat Med. 2003;9:1195–1201. 12910262 10.1038/nm912

[5] Cohen P, Frame S. The renaissance of GSK3. Nat Rev Mol Cell Biol. 2001;2:769–776. 11584304 10.1038/35096075

[6] Leroy K, Brion JP. Developmental expression and localization of glycogen synthase kinase-3beta in rat brain. J Chem Neuroanat. 1999;16:279–293. 10450875 10.1016/s0891-0618(99)00012-5

[7] Kelly S, Zhao H, Hua Sun G, Cheng D, Qiao Y, Luo J, et al Glycogen synthase kinase 3beta inhibitor Chir025 reduces neuronal death resulting from oxygen-glucose deprivation, glutamate excitotoxicity, and cerebral ischemia. Exp Neurol. 2004;188:378–386. 15246837 10.1016/j.expneurol.2004.04.004

[8] Guo W, Murthy AC, Zhang L, Johnson EB, Schaller EG, Allan AM, et al Inhibition of GSK3beta improves hippocampus-dependent learning and rescues neurogenesis in a mouse model of fragile X syndrome. Hum Mol Genet. 2012;21:681–691. 10.1093/hmg/ddr501 22048960 PMC3259018

[9] Lucas JJ, Hernandez F, Gomez-Ramos P, Moran MA, Hen R, Avila J. Decreased nuclear beta-catenin, tau hyperphosphorylation and neurodegeneration in GSK-3beta conditional transgenic mice. EMBO J. 2001;20:27–39. 11226152 10.1093/emboj/20.1.27 PMC140191

[10] Sirerol-Piquer M, Gomez-Ramos P, Hernandez F, Perez M, Moran MA, Fuster-Matanzo A, et al GSK3beta overexpression induces neuronal death and a depletion of the neurogenic niches in the dentate gyrus. Hippocampus. 2011;21:910–922. 10.1002/hipo.20805 20575007

[11] Lansberg MG, Bluhmki E, Thijs VN. Efficacy and safety of tissue plasminogen activator 3 to 4.5 hours after acute ischemic stroke: a metaanalysis. Stroke. 2009;40:2438–2441. 10.1161/STROKEAHA.109.552547 19478213 PMC2725521

[12] Kaur J, Zhao Z, Klein GM, Lo EH, Buchan AM. The neurotoxicity of tissue plasminogen activator? J Cereb Blood Flow Metab. 2004;24:945–963. 15356416 10.1097/01.WCB.0000137868.50767.E8

[13] Cai G, Liu B, Liu W, Tan X, Rong J, Chen X, et al Buyang Huanwu Decoction can improve recovery of neurological function, reduce infarction volume, stimulate neural proliferation and modulate VEGF and Flk1 expressions in transient focal cerebral ischaemic rat brains. J Ethnopharmacol. 2007;113:292–299. 17692486 10.1016/j.jep.2007.06.007

[14] Fan L, Wang K, Cheng B. Effects of buyang huanwu decoction on apoptosis of nervous cells and expressions of Bcl-2 and bax in the spinal cord of ischemia-reperfusion injury in rabbits. J Tradit Chin Med. 2006;26:153–156. 16817283

[15] Li XM, Bai XC, Qin LN, Huang H, Xiao ZJ, Gao TM. Neuroprotective effects of Buyang Huanwu Decoction on neuronal injury in hippocampus after transient forebrain ischemia in rats. Neurosci Lett. 2003;346:29–32. 12850540 10.1016/s0304-3940(03)00522-6

[16] Cheng YS, Cheng WC, Yao CH, Hsieh CL, Lin JG, Lai TY, et al Effects of buyang huanwu decoction on peripheral nerve regeneration using silicone rubber chambers. Am J Chin Med. 2001;29:423–432. 11789585 10.1142/S0192415X01000447

[17] Zheng GQ, Wang XM, Wang Y, Wang XT. Tau as a potential novel therapeutic target in ischemic stroke. J Cell Biochem. 2010;109:26–29. 10.1002/jcb.22408 19921714

[18] Wang HW, Liou KT, Wang YH, Lu CK, Lin YL, Lee IJ, et al Deciphering the neuroprotective mechanisms of Bu-yang Huan-wu decoction by an integrative neurofunctional and genomic approach in ischemic stroke mice. J Ethnopharmacol. 2011;138:22–33. 10.1016/j.jep.2011.06.033 21784143

[19] Chen HJ, Shen YC, Lin CY, Tsai KC, Lu CK, Shen CC, et al Metabolomics study of Buyang Huanwu Tang Decoction in ischemic stroke mice by ^1^H NMR. Metabolomics. 2012;8:974–984. 10.1007/s11306-011-0394-0

[20] Hao CZ, Wu F, Shen J, Lu L, Fu DL, Liao WJ, et al Clinical efficacy and safety of buyang huanwu decoction for acute ischemic stroke: a systematic review and meta-analysis of 19 randomized controlled trials. Evid Based Complement Alternat Med. 2012;2012:630124 10.1155/2012/630124 23193426 PMC3491750

[21] Cho WC. Application of proteomics in Chinese medicine research. Am J Chin Med. 2007;35:911–922. 18186577 10.1142/S0192415X07005375

[22] Gao M, Deng C, Lin S, Hu F, Tang J, Yao N, et al Recent developments and contributions from Chinese scientists in multidimensional separations for proteomics and traditional Chinese medicines. J Sep Sci. 2007;30:785–791. 17536722 10.1002/jssc.200600372 PMC7167053

[23] Ulrich-Merzenich G, Zeitler H, Jobst D, Panek D, Vetter H, Wagner H. Application of the "-Omic-" technologies in phytomedicine. Phytomedicine. 2007;14:70–82. 17188482 10.1016/j.phymed.2006.11.011

[24] DeSouza L, Diehl G, Rodrigues MJ, Guo J, Romaschin AD, Colgan TJ, et al Search for cancer markers from endometrial tissues using differentially labeled tags iTRAQ and cICAT with multidimensional liquid chromatography and tandem mass spectrometry. J Proteome Res. 2005;4:377–386. 15822913 10.1021/pr049821j

[25] Liu X, Guo DA. Application of proteomics in the mechanistic study of traditional Chinese medicine. Biochem Soc Trans. 2011;39:1348–1352. 10.1042/BST0391348 21936813

[26] Stroke Therapy Academic Industry Roundtable. Recommendations for standards regarding preclinical neuroprotective and restorative drug development. Stroke 1999, 30, 2752–2758. 10583007 10.1161/01.str.30.12.2752

[27] Kunz A, Abe T, Hochrainer K, Shimamura M, Anrather J, Racchumi G, et al Nuclear factor-kappaB activation and postischemic inflammation are suppressed in CD36-null mice after middle cerebral artery occlusion. J Neurosci. 2008;28:1649–1658. 10.1523/JNEUROSCI.5205-07.2008 18272685 PMC2588435

[28] Reagan-Shaw S, Nihal M, Ahmad N. Dose translation from animal to human studies revisited. FASEB J. 2008;22:659–661. 17942826 10.1096/fj.07-9574LSF

[29] Chern CM, Liao JF, Wang YH, Shen YC. Melatonin ameliorates neural function by promoting endogenous neurogenesis through the MT2 melatonin receptor in ischemic-stroke mice. Free Radic Biol Med. 2012;52:1634–1647. 10.1016/j.freeradbiomed.2012.01.030 22330064

[30] Chen CJ, Tseng MC, Lin HJ, Lin TW, Chen YR. Visual indicator for surfactant abundance in MS-based membrane and general proteomics applications. Anal Chem. 2010;82:8283–8290. 10.1021/ac1017937 20828166

[31] Chang WH, Lee CY, Lin CY, Chen WY, Chen MC, Tzou WS, et al UniQua: a universal signal processor for MS-based qualitative and quantitative proteomics applications. Anal Chem. 2013;85:890–897. 10.1021/ac1017937 23237057

[32] Keller A, Nesvizhskii AI, Kolker E, Aebersold R. Empirical statistical model to estimate the accuracy of peptide identifications made by MS/MS and database search. Anal Chem. 2002;74:5383–5392. 12403597 10.1021/ac025747h

[33] Huang da W, Sherman BT, Lempicki RA. Systematic and integrative analysis of large gene lists using DAVID bioinformatics resources. Nat Protoc. 2009;4:44–57. 10.1038/nprot.2008.211 19131956

[34] Lindvall O, Kokaia Z. Stem cell research in stroke: how far from the clinic? Stroke. 2011;42:2369–2375. 10.1161/STROKEAHA.110.599654 21757669

[35] Tissue plasminogen activator for acute ischemic stroke. The National Institute of Neurological Disorders and Stroke rt-PA Stroke Study Group. N Engl J Med. 1995;333:1581–1587. 7477192 10.1056/NEJM199512143332401

[36] Whiteley W, Chong WL, Sengupta A, Sandercock P. Blood markers for the prognosis of ischemic stroke: a systematic review. Stroke. 2009;40:e380–389. 10.1161/STROKEAHA.108.528752 19286602

[37] Prajapati KD, Sharma SS, Roy N. Upregulation of albumin expression in focal ischemic rat brain. Brain Res. 2010;1327:118–124. 10.1016/j.brainres.2010.02.063 20193666

[38] Ryu JK, Davalos D, Akassoglou K. Fibrinogen signal transduction in the nervous system. J Thromb Haemost. 2009;7 Suppl 1:151–154. 10.1111/j.1538-7836.2009.03438.x 19630789 PMC2888044

[39] Shimamura N, Matchett G, Yatsushige H, Calvert JW, Ohkuma H, Zhang J. Inhibition of integrin alphavbeta3 ameliorates focal cerebral ischemic damage in the rat middle cerebral artery occlusion model. Stroke. 2006;37:1902–1909. 16741177 10.1161/01.STR.0000226991.27540.f2

[40] Altamura C, Squitti R, Pasqualetti P, Gaudino C, Palazzo P, Tibuzzi F, et al Ceruloplasmin/Transferrin system is related to clinical status in acute stroke. Stroke. 2009;40:1282–1288. 10.1161/STROKEAHA.108.536714 19228837

[41] Datta A, Jingru Q, Khor TH, Teo MT, Heese K, Sze SK. Quantitative neuroproteomics of an in vivo rodent model of focal cerebral ischemia/reperfusion injury reveals a temporal regulation of novel pathophysiological molecular markers. J Proteome Res. 2011;10:5199–5213. 10.1021/pr200673y 21950801

[42] Jin R, Yang G, Li G. Molecular insights and therapeutic targets for blood-brain barrier disruption in ischemic stroke: critical role of matrix metalloproteinases and tissue-type plasminogen activator. Neurobiol Dis. 2010;38:376–385. 10.1016/j.nbd.2010.03.008 20302940 PMC2862862

[43] Govek EE, Newey SE, Van Aelst L. The role of the Rho GTPases in neuronal development. Genes Dev. 2005;19:1–49. 15630019 10.1101/gad.1256405

[44] Liu J, Wang LN. Gamma aminobutyric acid (GABA) receptor agonists for acute stroke. Cochrane Database Syst Rev. 2013;2:CD009622 10.1002/14651858.CD009622.pub2 23450607

[45] Niswender CM, Conn PJ. Metabotropic glutamate receptors: physiology, pharmacology, and disease. Annu Rev Pharmacol Toxicol. 2010;50:295–322. 10.1146/annurev.pharmtox.011008.145533 20055706 PMC2904507

[46] Li H, Zhang N, Sun G, Ding S. Inhibition of the group I mGluRs reduces acute brain damage and improves long-term histological outcomes after photothrombosis-induced ischaemia. ASN Neuro. 2013;5:195–207. 10.1042/AN20130002 23772679 PMC3786425

[47] Nochi R, Kato T, Kaneko J, Itou Y, Kuribayashi H, Fukuda S, et al Involvement of metabotropic glutamate receptor 5 signaling in activity-related proliferation of adult hippocampal neural stem cells. Eur J Neurosci. 2012;36:2273–2283. 10.1111/j.1460-9568.2012.08128.x 22591399

[48] Bao WL, Williams AJ, Faden AI, Tortella FC. Selective mGluR5 receptor antagonist or agonist provides neuroprotection in a rat model of focal cerebral ischemia. Brain Res. 2001;922:173–179. 11743947 10.1016/s0006-8993(01)03062-1

[49] Zhang M, Li WB, Geng JX, Li QJ, Sun XC, Xian XH, et al The upregulation of glial glutamate transporter-1 participates in the induction of brain ischemic tolerance in rats. J Cereb Blood Flow Metab. 2007;27:1352–1368. 17228332 10.1038/sj.jcbfm.9600441

[50] Beschorner R, Simon P, Schauer N, Mittelbronn M, Schluesener HJ, Trautmann K, et al Reactive astrocytes and activated microglial cells express EAAT1, but not EAAT2, reflecting a neuroprotective potential following ischaemia. Histopathology. 2007;50:897–910. 17543080 10.1111/j.1365-2559.2007.02703.x

[51] Ziegler G, Prinz V, Albrecht MW, Harhausen D, Khojasteh U, Nacken W, et al Mrp-8 and -14 mediate CNS injury in focal cerebral ischemia. Biochim Biophys Acta. 2009;1792:1198–1204. 10.1016/j.bbadis.2009.10.003 19835955

[52] Marchi N, Fazio V, Cucullo L, Kight K, Masaryk T, Barnett G, et al Serum transthyretin monomer as a possible marker of blood-to-CSF barrier disruption. J Neurosci. 2003;23:1949–1955. 12629200 10.1523/JNEUROSCI.23-05-01949.2003 PMC6741971

[53] Korshunova I, Caroni P, Kolkova K, Berezin V, Bock E, Walmod PS. Characterization of BASP1-mediated neurite outgrowth. J Neurosci Res. 2008;86:2201–2213. 10.1002/jnr.21678 18438920

[54] Kim N, Lee Y, Kim H, Joo H, Youm JB, Park WS, et al Potential biomarkers for ischemic heart damage identified in mitochondrial proteins by comparative proteomics. Proteomics. 2006;6:1237–1249. Proteomics. 2006;6:1237–1249. 16402359 10.1002/pmic.200500291

[55] Imhof A, Charnay Y, Vallet PG, Aronow B, Kovari E, French LE, et al Sustained astrocytic clusterin expression improves remodeling after brain ischemia. Neurobiol Dis. 2006;22:274–283. 16473512 10.1016/j.nbd.2005.11.009

[56] Hotta A, Inatome R, Yuasa-Kawada J, Qin Q, Yamamura H, Yanagi S. Critical role of collapsin response mediator protein-associated molecule CRAM for filopodia and growth cone development in neurons. Mol Biol Cell. 2005;16:32–39. 15509652 10.1091/mbc.E04-08-0679 PMC539149

[57] Liu PC, Yang ZJ, Qiu MH, Zhang LM, Sun FY. Induction of CRMP-4 in striatum of adult rat after transient brain ischemia. Acta Pharmacol Sin. 2003;24:1205–1211. 14653945

[58] Armogida M, Nistico R, Mercuri NB. Therapeutic potential of targeting hydrogen peroxide metabolism in the treatment of brain ischaemia. Br J Pharmacol. 2012;166:1211–1224. 10.1111/j.1476-5381.2012.01912.x 22352897 PMC3417441

[59] Gold AB, Herrmann N, Lanctot KL. Lithium and its neuroprotective and neurotrophic effects: potential treatment for post-ischemic stroke sequelae. Curr Drug Targets. 2011;12:243–255. 20863277 10.2174/138945011794182764

[60] Zhao H, Shimohata T, Wang JQ, Sun G, Schaal DW, Sapolsky RM, et al Akt contributes to neuroprotection by hypothermia against cerebral ischemia in rats. J Neurosci. 2005;25:9794–9806. 16237183 10.1523/JNEUROSCI.3163-05.2005 PMC6725740

[61] Fang X, Yu S, Tanyi JL, Lu Y, Woodgett JR, Mills GB. Convergence of multiple signaling cascades at glycogen synthase kinase 3: Edg receptor-mediated phosphorylation and inactivation by lysophosphatidic acid through a protein kinase C-dependent intracellular pathway. Mol Cell Biol. 2002;22:2099–2110. 11884598 10.1128/MCB.22.7.2099-2110.2002 PMC133668

[62] Song B, Lai B, Zheng Z, Zhang Y, Luo J, Wang C, et al Inhibitory phosphorylation of GSK-3 by CaMKII couples depolarization to neuronal survival. J Biol Chem. 2010;285:41122–41134. 10.1074/jbc.M110.130351 20841359 PMC3003410

[63] Nelson TJ, Alkon DL. Neuroprotective versus tumorigenic protein kinase C activators. Trends Biochem Sci. 2009;34:136–145. 10.1016/j.tibs.2008.11.006 19233655

[64] Tan Z, Turner RC, Leon RL, Li X, Hongpaisan J, Zheng W, et al Bryostatin improves survival and reduces ischemic brain injury in aged rats after acute ischemic stroke. Stroke. 2013;44:3490–3497. 10.1161/STROKEAHA.113.002411 24172582 PMC4041549

[65] Nakatomi H, Kuriu T, Okabe S, Yamamoto S, Hatano O, Kawahara N, et al Regeneration of hippocampal pyramidal neurons after ischemic brain injury by recruitment of endogenous neural progenitors. Cell. 2002;110:429–441. . 12202033 10.1016/s0092-8674(02)00862-0

