# Supplementary figures and images for: Multiplex Brain Proteomic Analysis Revealed the Molecular Therapeutic Effects of Buyang Huanwu Decoction on Cerebral Ischemic Stroke Mice
Source: PLoS One. 2015 Oct 22;10(10):e0140823. doi: 10.1371/journal.pone.0140823 (PMC4619651; doi:10.1371/journal.pone.0140823)

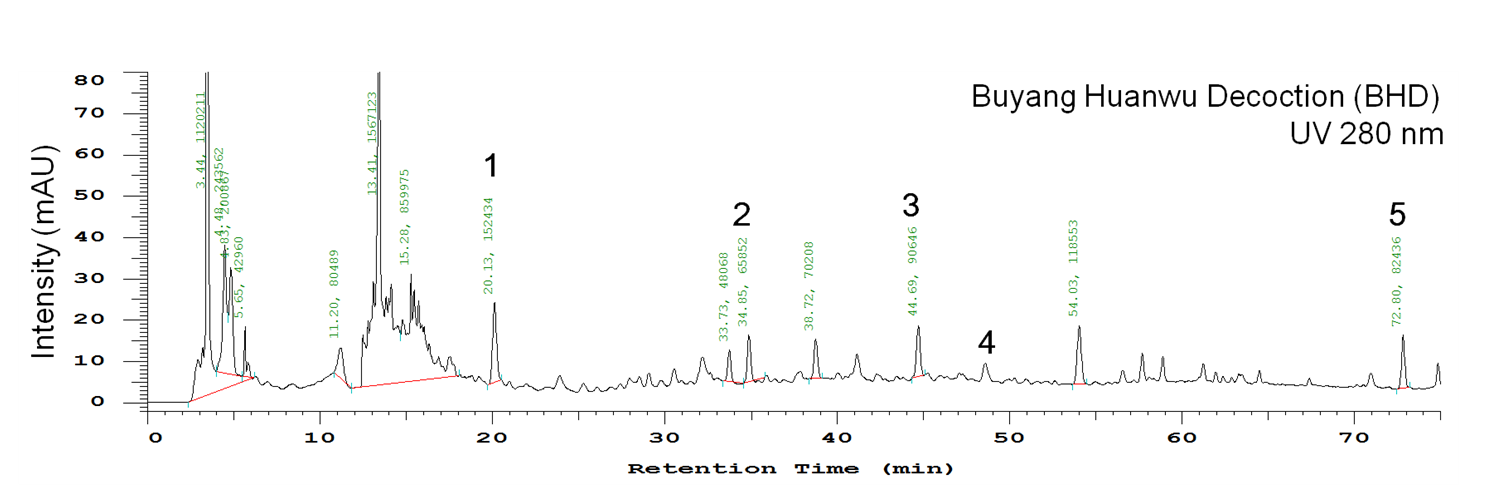

Supplement: S1 Fig — (TIF) [file pone.0140823.s001.tif]

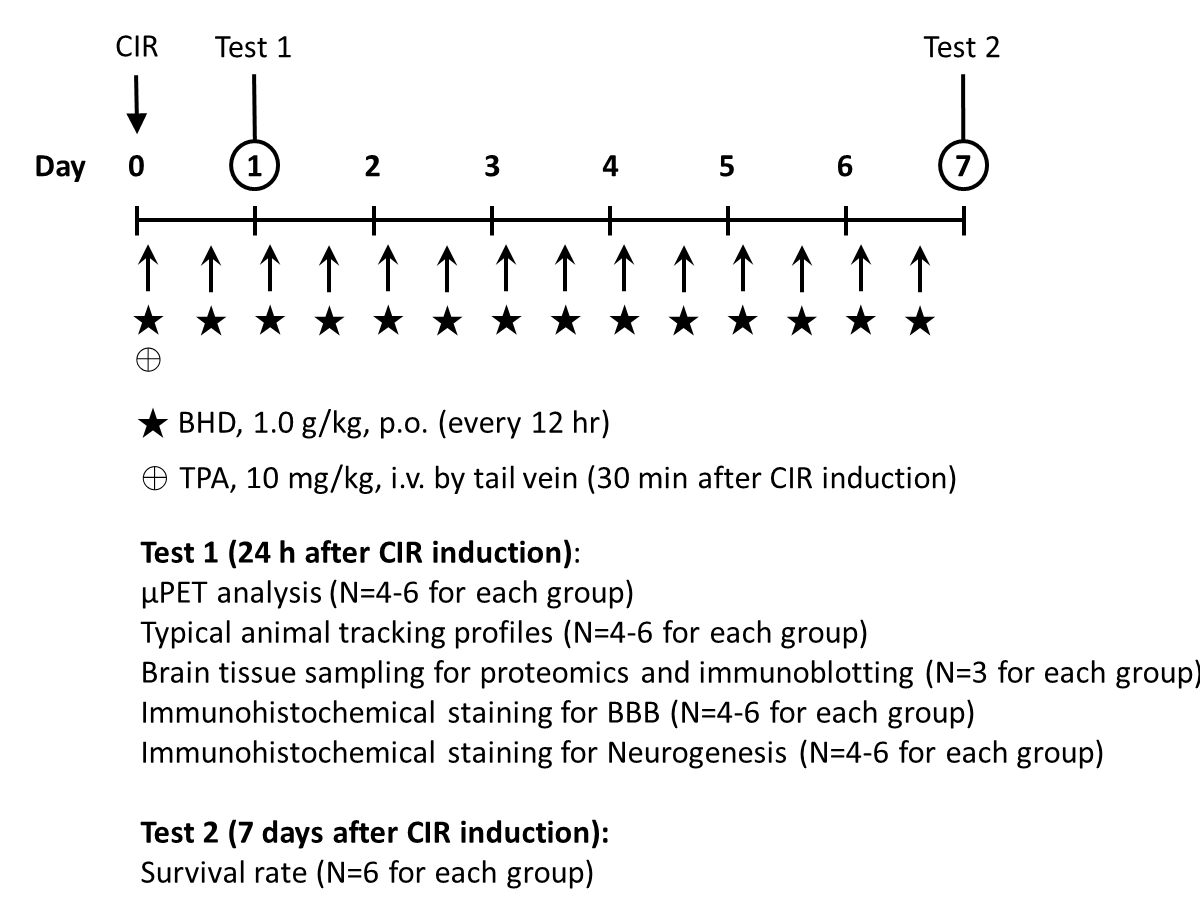

Supplement: S2 Fig — (TIF) [file pone.0140823.s002.tif]

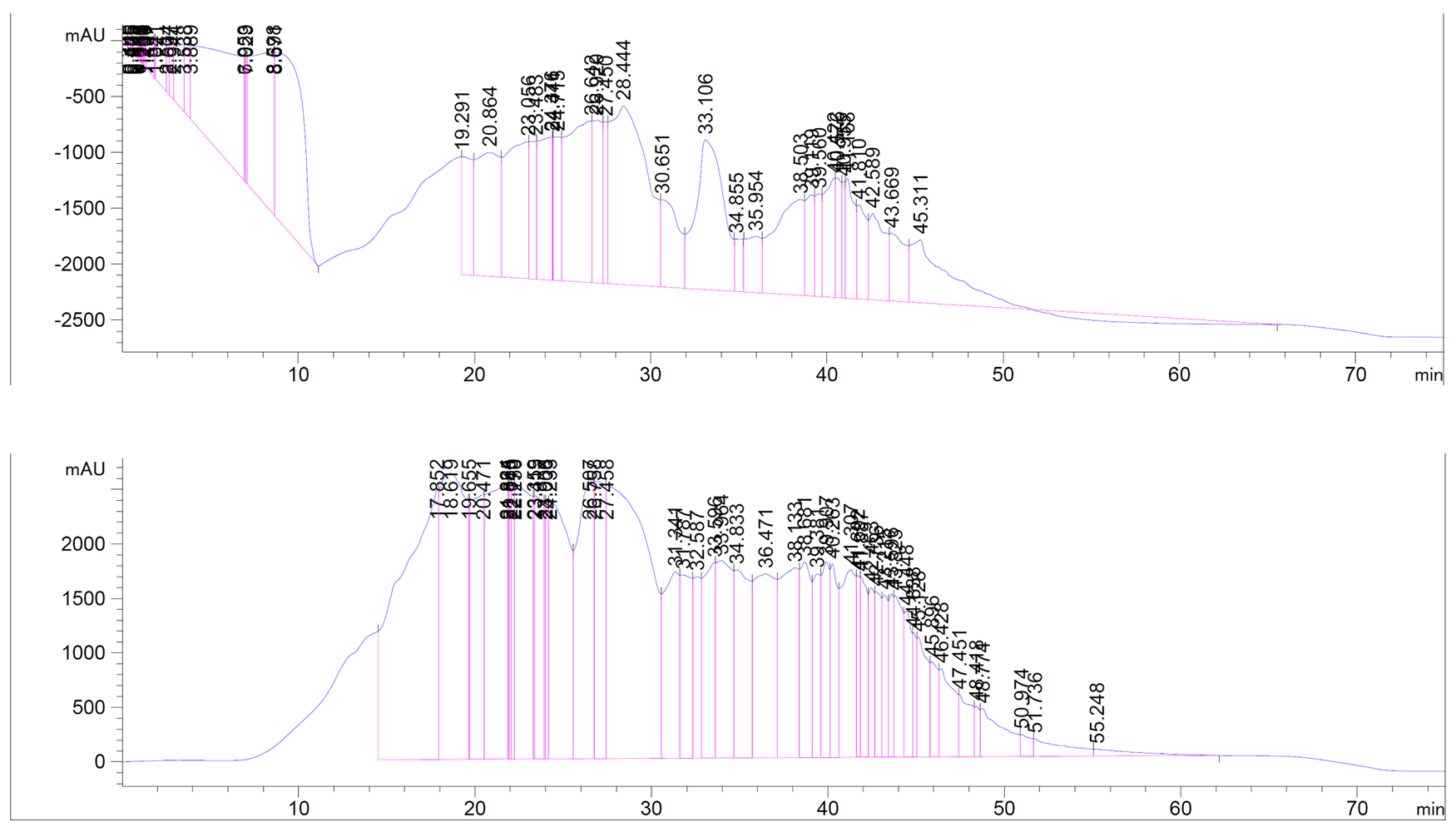

Supplement: S3 Fig — (TIF) [file pone.0140823.s003.tif]
