# Supplementary material for: Multiplex Brain Proteomic Analysis Revealed the Molecular Therapeutic Effects of Buyang Huanwu Decoction on Cerebral Ischemic Stroke Mice
Source: PLoS One. 2015 Oct 22;10(10):e0140823. doi: 10.1371/journal.pone.0140823 (PMC4619651; doi:10.1371/journal.pone.0140823)
Supplement: S1 Table — (DOC) [file pone.0140823.s004.doc]

Table S1. Antibodies used in this experiment.

| Target Protein | Cat. NO. |
| --- | --- |
| **Cell Signaling**^1^ | |
| pS^473^-Akt | 9271 |
| CaMK II α/β | 4436 |
| pS^21/9^-GSK-3α/β | 9327 |
| pT^638/641^-PKCα/β_II_ | 9375 |
| pT^410/403^-PKCζ/λ | 9378 |
| **Millipore**^2^ | |
| pS^729^-PKCε | 06-821 |
| **Bioss**^3^ | |
| Bdh | bs-5041R |
| Gap43 | bs-0154R |
| Mag | bs-0257R |
| pS^202^-Tau | bs-11240R |
| **Abgent^4^** | |
| Actn1 | AP8512a |
| Aplp1 | AM2143b |
| Cntn1 | AP9490c |
| ^1^Cell Signaling Technolohy, Inc. (Danvers, MA, USA) | |
| ^2^Merck Millipore (Billerica, MA, USA) | |
| ^3^Bioss Inc. (Woburn, MA, USA) | |
| ^4^Abgent Inc. (San Diego, CA, USA) | |
